# Hepatic FASN deficiency differentially affects nonalcoholic fatty liver disease and diabetes in mouse obesity models

**DOI:** 10.1172/jci.insight.161282

**Published:** 2023-09-08

**Authors:** Toshiya Matsukawa, Takashi Yagi, Tohru Uchida, Mashito Sakai, Masaru Mitsushima, Takao Naganuma, Hiroyuki Yano, Yuka Inaba, Hiroshi Inoue, Keisuke Yanagida, Masaaki Uematsu, Kazuki Nakao, Harumi Nakao, Atsu Aiba, Yoji Nagashima, Tetsuya Kubota, Naoto Kubota, Yoshihiko Izumida, Naoya Yahagi, Hiroyuki Unoki-Kubota, Yasushi Kaburagi, Shun-ichiro Asahara, Yoshiaki Kido, Hideo Shindou, Michiko Itoh, Yoshihiro Ogawa, Shiro Minami, Yasuo Terauchi, Kazuyuki Tobe, Kohjiro Ueki, Masato Kasuga, Michihiro Matsumoto

**Affiliations:** 1 Department of Molecular Metabolic Regulation, Diabetes Research Center, Research Institute, National Center for Global Health and Medicine (NCGM), Tokyo, Japan.; 2 Department of Bioregulation, Institute for Advanced Medical Sciences, Nippon Medical School, Kawasaki, Kanagawa, Japan.; 3 Department of Nutrition Management, Faculty of Health Science, Hyogo University, Kakogawa, Hyogo, Japan.; 4 Metabolism and Nutrition Research Unit, Institute for Frontier Science Initiative, and; 5 Department of Physiology and Metabolism, Graduate School of Medical Sciences, Kanazawa University, Kanazawa, Ishikawa, Japan.; 6 Department of Lipid Life Science, NCGM, Tokyo, Japan.; 7 Institute of Experimental Animal Sciences, Osaka University Graduate School of Medicine, Osaka, Japan.; 8 Laboratory of Animal Resources, Center for Disease Biology and Integrative Medicine, Graduate School of Medicine and Faculty of Medicine, The University of Tokyo, Tokyo, Japan.; 9 Department of Surgical Pathology, School of Medicine, Tokyo Women’s Medical University, Tokyo, Japan.; 10 Department of Diabetes and Metabolic Diseases, Graduate School of Medicine and Faculty of Medicine, The University of Tokyo, Tokyo, Japan.; 11 Division of Diabetes and Metabolism, The Institute of Medical Science, Asahi Life Foundation, Tokyo, Japan.; 12 Department of Clinical Nutrition, National Institutes of Biomedical Innovation, Health, and Nutrition (NIBIOHN), Tokyo, Japan.; 13 Department of Clinical Nutrition Therapy, The University of Tokyo, Tokyo, Japan.; 14 Nutrigenomics Research Group, Faculty of Medicine, University of Tsukuba, Tsukuba, Ibaraki, Japan.; 15 Department of Diabetic Complications, Diabetes Research Center, Research Institute, NCGM, Tokyo, Japan.; 16 Division of Diabetes and Endocrinology, Department of Internal Medicine, and; 17 Division of Medical Chemistry, Department of Metabolism and Disease, Kobe University Graduate School of Health Sciences, Kobe, Hyogo, Japan.; 18 Department of Medical Lipid Science, Graduate School of Medicine, The University of Tokyo, Tokyo, Japan.; 19 Department of Metabolic Syndrome and Nutritional Science, Research Institute of Environmental Medicine, Nagoya University, Nagoya, Japan.; 20 Department of Medicine and Bioregulatory Science, Graduate School of Medical Sciences, Kyushu University, Fukuoka, Japan.; 21 Department of Endocrinology and Metabolism, Yokohama City University Graduate School of Medicine, Yokohama, Kanagawa, Japan.; 22 First Department of Internal Medicine, University of Toyama, Toyama-shi, Toyama, Japan.; 23 Department of Molecular Diabetic Medicine, Diabetes Research Center, Research Institute, NCGM, Tokyo, Japan.; 24 The Institute of Medical Science, Asahi Life Foundation, Tokyo, Japan.; 25 Course of Advanced and Specialized Medicine, Juntendo University Graduate School of Medicine, Tokyo, Japan.

**Keywords:** Hepatology, Metabolism, Diabetes, Molecular pathology, Obesity

## Abstract

Nonalcoholic fatty liver disease (NAFLD) and type 2 diabetes are interacting comorbidities of obesity, and increased hepatic de novo lipogenesis (DNL), driven by hyperinsulinemia and carbohydrate overload, contributes to their pathogenesis. Fatty acid synthase (FASN), a key enzyme of hepatic DNL, is upregulated in association with insulin resistance. However, the therapeutic potential of targeting FASN in hepatocytes for obesity-associated metabolic diseases is unknown. Here, we show that hepatic FASN deficiency differentially affects NAFLD and diabetes depending on the etiology of obesity. Hepatocyte-specific ablation of FASN ameliorated NAFLD and diabetes in melanocortin 4 receptor–deficient mice but not in mice with diet-induced obesity. In leptin-deficient mice, FASN ablation alleviated hepatic steatosis and improved glucose tolerance but exacerbated fed hyperglycemia and liver dysfunction. The beneficial effects of hepatic FASN deficiency on NAFLD and glucose metabolism were associated with suppression of DNL and attenuation of gluconeogenesis and fatty acid oxidation, respectively. The exacerbation of fed hyperglycemia by FASN ablation in leptin-deficient mice appeared attributable to impairment of hepatic glucose uptake triggered by glycogen accumulation and citrate-mediated inhibition of glycolysis. Further investigation of the therapeutic potential of hepatic FASN inhibition for NAFLD and diabetes in humans should thus consider the etiology of obesity.

## Graphical abstract

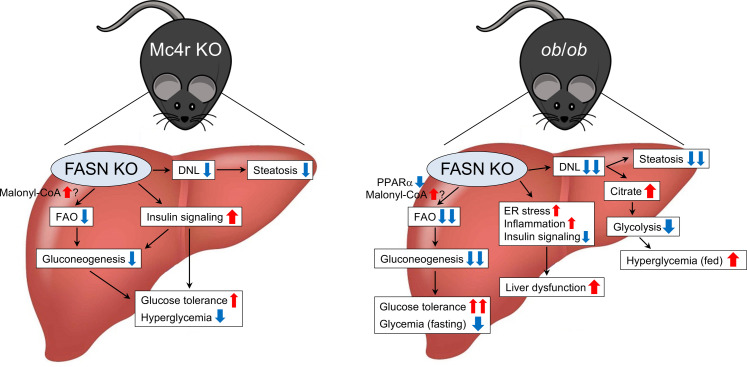

## Introduction

Nonalcoholic fatty liver disease (NAFLD) is characterized by hepatic lipid accumulation (hepatic steatosis) in the absence of excessive alcohol consumption and has a global prevalence of 25% (1). It encompasses conditions ranging from nonalcoholic fatty liver (NAFL, also known as isolated steatosis) to nonalcoholic steatohepatitis (NASH). NAFL may progress to NASH, which is characterized histologically by steatosis, hepatocyte injury, and lobular inflammation. NASH in turn can lead to severe outcomes such as liver cirrhosis and, occasionally, hepatocellular carcinoma (2). NAFLD is strongly associated with both obesity and type 2 diabetes mellitus (T2D), with a prevalence of up to 80% and 64% in individuals with these conditions, respectively (3, 4). Individuals with T2D thus have a >2-fold higher prevalence of NAFLD (5), and those with NAFLD have a >2-fold increased risk of T2D (6). This bidirectional interaction between NAFLD and T2D is mediated by obesity-associated metabolic abnormalities such as overfeeding, insulin resistance, hyperinsulinemia, and hyperglycemia.

In the liver, triglyceride is produced by esterification of glycerol with fatty acids that are predominantly derived from dietary fat, adipose lipolysis, and hepatic de novo lipogenesis (DNL). Depletion of triglyceride in the liver is determined by catabolism and export, specifically, by mitochondrial fatty acid oxidation (FAO, or β-oxidation) and secretion of VLDL, respectively. Hepatic steatosis develops when triglyceride production exceeds triglyceride depletion (7). Obese individuals with NAFLD have a >3-fold higher rate of DNL and a 2-fold higher proportion of hepatic triglyceride produced as a result of DNL — rather than from free fatty acids (FFAs) derived from adipose lipolysis or the diet — compared with those without NAFLD (8). Leptin-deficient (*ob/ob*) mice, which develop obesity, hepatic steatosis, and diabetes as a result of hyperphagia and insulin resistance, also have a 10-fold higher rate of hepatic DNL compared with control mice (9). Enhanced DNL is thus a central abnormality of NAFLD in both humans and rodents.

DNL is mediated by 3 lipogenic enzymes — ATP citrate lyase (ACLY), which produces acetyl-CoA; acetyl-CoA carboxylase (ACC), which produces malonyl-CoA from acetyl-CoA; and fatty acid synthase (FASN) — and results in the generation of palmitate from TCA cycle–derived citrate, thereby linking carbohydrate and lipid metabolism. Hyperinsulinemia and carbohydrate overload cooperatively promote hepatic DNL in obese mice. Hyperinsulinemia, which results from insulin resistance, promotes transcriptional induction of SREBP1c, a key transcription factor in the regulation of lipogenic gene expression (10), through activation of a signaling pathway including the insulin receptor, insulin receptor substrate 1, PI3K, and either Akt or atypical PKC in hepatocytes (11–15), whereas insulin also promotes proteolytic activation of SREBP1c (16). On the other hand, carbohydrates serve as an acetyl-CoA donor and activate carbohydrate response element–binding protein (ChREBP), another lipogenic transcription factor, in the liver (17). ChREBP induces the transcription of genes related to glycolysis (including that for pyruvate kinase liver/red blood cell [*Pklr*]), fructolysis (including those for ketohexokinase and aldolase B [*Aldob*]), and DNL (18, 19). Activation of the lipogenic program by SREBP1c and ChREBP in the liver thus contributes to the development of NAFLD. Enhanced hepatic DNL results in the accumulation of lipid moieties including palmitate, diacylglycerol, and ceramide, which in turn triggers insulin resistance through antagonism of insulin signaling (20–22). Hepatic DNL and insulin resistance therefore form a vicious cycle that contributes to the pathogenesis of both NAFLD and T2D (23), with inhibition of hepatic DNL being a potential therapeutic strategy for these diseases.

FASN is a key lipogenic enzyme that ultimately synthesizes palmitate with malonyl-CoA as a 2-carbon donor (24–26). Hepatic expression of FASN is upregulated by both ChREBP and SREBP1c (18) in obese diabetic mice with NAFLD (27–29). Nonobese mice with hepatocyte-specific FASN deficiency (HKO mice) that were fed a zero-fat diet (ZFD) containing a high proportion of sucrose (62% by weight), but not those fed normal chow, developed aggravated NAFLD (despite impairment of DNL) as well as hypoglycemia due to impaired FAO (30). In contrast, pharmacological inhibition of FASN in leptin receptor–deficient (*db/db*) mice ameliorated fed hyperglycemia, but not hepatic steatosis, in association with a reduction in food intake and BW gain, with these findings having cast doubt on the liver specificity of the drug action (31, 32). The therapeutic potential of targeting hepatic FASN in obesity-related metabolic diseases has thus remained unclear.

With the use of HKO mice with diet-induced or genetic obesity, we now show that hepatic FASN deficiency differentially affects NAFLD and diabetes in a manner dependent on the etiology of obesity. Hepatic FASN deficiency thus ameliorated NAFLD and diabetes in melanocortin 4 receptor–deficient (Mc4r-KO) mice but not in mice fed a high-fat diet (HFD). In *ob/ob* mice, hepatic FASN deficiency ameliorated hepatic steatosis and improved glucose tolerance to a greater extent than in Mc4r-KO mice, but it exacerbated fed hyperglycemia and liver dysfunction. Amelioration of NAFLD was associated with suppression of hepatic DNL, whereas improved glucose metabolism appeared to result from suppression of both hepatic gluconeogenesis and FAO, with these latter effects being mediated by different mechanisms in Mc4r-KO and leptin-deficient mice. The exacerbation of fed hyperglycemia by hepatic FASN ablation in leptin-deficient mice appeared attributable to impairment of hepatic glucose uptake triggered by hepatic glycogen accumulation and citrate-mediated inhibition of glycolysis. Our findings thus indicate that the therapeutic efficacy of hepatic FASN inhibition for NAFLD and T2D might be determined by the etiology of obesity, which should be taken into account in follow-up investigations in humans.

## Results

### Generation of HKO mice and their analysis while maintained on a normal chow diet.

**Figure 1:**
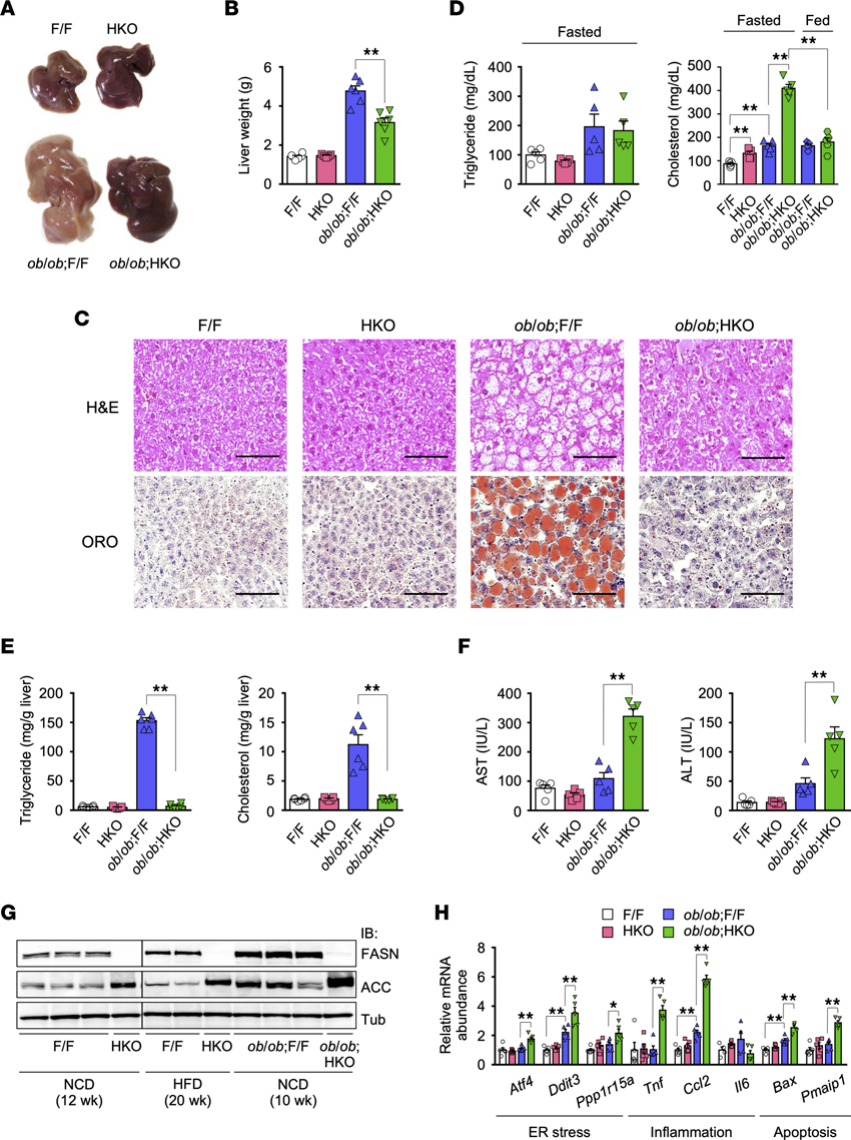
Hepatic FASN deficiency in *ob/ob* mice ameliorates hepatic steatosis but exacerbates liver dysfunction. (**A**–**C**) Macroscopic appearance of the liver (**A**), liver weight (**B**), and H&E and Oil Red O (ORO) staining of liver sections (**C**) for 10-week-old NCD-fed F/F, HKO, *ob/ob* F/F, and *ob/ob* HKO mice. Images in **A** and **C** are representative of 5 mice per group. Scale bars, 100 μm (**C**). (**D** and **E**) Triglyceride and cholesterol levels in plasma (**D**) and the liver (**E**) of 10-week-old mice either deprived of food overnight (plasma) or in the fed state (plasma and liver). (**F**) Plasma aspartate aminotransaminase (AST) and alanine aminotransaminase (ALT) levels in 10-week-old mice. (**G**) Immunoblot analysis of FASN and ACC in the liver of NCD- or HFD-fed mice at the indicated ages. α-Tubulin (Tub) was examined as a loading control. The lanes are from the same gel but were noncontiguous. Each lane corresponds to 1 mouse, and the blots are representative of 2 independent experiments. (**H**) RT-qPCR analysis of gene expression related to ER stress, inflammation, or apoptosis in the liver of 10-week-old mice in the fed state. All quantitative data are means + SEM (*n* = 5 or 6 mice). *P < 0.05, **P < 0.01 (1-way ANOVA followed by Tukey’s in **B**, **D**, **E**, and **F** or Bonferroni’s multiple-comparison test in **H**).

To obtain HKO mice, we first generated mice heterozygous for a floxed *Fasn* allele (F/+ mice) by homologous recombination (Supplemental Figure 1, A and B; supplemental material available online with this article; https://doi.org/10.1172/jci.insight.161282DS1; full unedited blots also available online) and then crossed mice homozygous for the floxed *Fasn* allele (F/F mice, which were studied as controls) with those expressing Cre recombinase under the control of the mouse albumin gene promoter (33). HKO mice were born at the expected Mendelian frequency and were indistinguishable from WT and F/F mice in gross appearance. Reverse transcription quantitative PCR (RT-qPCR) analysis and immunoblot analysis revealed that the hepatic abundance of *Fasn* mRNA and FASN protein decreased by ~75% and ~95%, respectively, in HKO mice compared with WT or F/F mice (Supplemental Figure 1, C and D). HKO mice maintained on a normal chow diet (NCD) showed no difference in BW, blood glucose, or plasma insulin concentrations in the fed state or blood glucose levels during an intraperitoneal glucose tolerance test (IPGTT) or insulin tolerance test (ITT) compared with F/F mice (Supplemental Figure 2). The gross appearance, weight, and histology of the liver; plasma and hepatic lipid concentrations (with the exception of a higher plasma cholesterol level in the fasted state in the mutant mice); and plasma transaminase levels were also indistinguishable between the 2 genotypes (Figure 1, A–F), essentially consistent with previous findings for NCD-fed HKO animals (30).

### Hepatic FASN deficiency in ob/ob mice ameliorates hepatic steatosis but exacerbates liver dysfunction.

To evaluate the therapeutic potential of targeting hepatic FASN for NAFLD and diabetes, we initially generated HKO and F/F mice with HFD-induced obesity. Immunoblot analysis showed that hepatic expression of FASN was slightly increased (1.8-fold) in HFD-fed F/F mice compared with NCD-fed F/F mice (Figure 1G). HFD-fed HKO and F/F mice were indistinguishable in terms of BW, 24-hour food intake, liver and fat weight, blood glucose and plasma insulin concentrations in the fed state, insulin sensitivity and glucose tolerance as assessed by metabolic tests, and hepatic triglyceride content, though the hepatic cholesterol level was increased in the mutant mice (Supplemental Figure 3). These results were thus indicative of only a minor contribution of hepatocyte FASN to hepatic steatosis and changes in glucose metabolism in mice fed an HFD, consistent with the finding that HFD intake promotes hepatic reesterification of fatty acids but not DNL (34).

Given that hepatic DNL is promoted by hyperinsulinemia and carbohydrate overfeeding, hyperinsulinemia alone in HFD-fed mice might be insufficient to promote hepatic DNL. To maximize the effect of hepatic FASN deficiency, we therefore next studied *ob/ob* mice fed an NCD, which develop hyperphagic obesity associated with hyperinsulinemia. Hepatic FASN expression was markedly upregulated in F/F mice on the *ob/ob* genetic background (*ob/ob* F/F mice) compared with NCD- or HFD-fed F/F mice (Figure 1G) or with *ob*/+ F/F mice (data not shown). The *ob/ob* F/F mice had pale and enlarged livers compared with NCD-fed F/F and HKO mice at 10 weeks of age (Figure 1, A and B). H&E and Oil Red O staining of liver sections (Figure 1C) as well as measurement of plasma and hepatic lipid levels (Figure 1, D and E) and plasma transaminase levels (Figure 1F) revealed the development of marked hepatic steatosis and liver dysfunction in *ob/ob* F/F mice. This steatosis was virtually eliminated in *ob/ob* HKO mice (Figure 1, A–C, and E), whereas plasma cholesterol (Figure 1D) and transaminase (Figure 1F) levels were further increased compared with *ob/ob* F/F mice. This increase in plasma cholesterol level was apparent in the fasted state but not in the fed state (Figure 1D). The increase in transaminase levels was associated with upregulation of the expression of genes related to ER stress, inflammation, and apoptosis in the liver (Figure 1H), suggestive of a role for these processes in the enhancement of liver dysfunction by FASN deficiency. These findings thus showed that hepatic FASN deficiency ameliorates hepatic steatosis but exacerbates liver dysfunction in *ob/ob* mice.

### Hepatic FASN deficiency in ob/ob mice improves glucose tolerance and confers relative fasting hypoglycemia but exacerbates hyperglycemia in the fed state.

**Figure 2:**
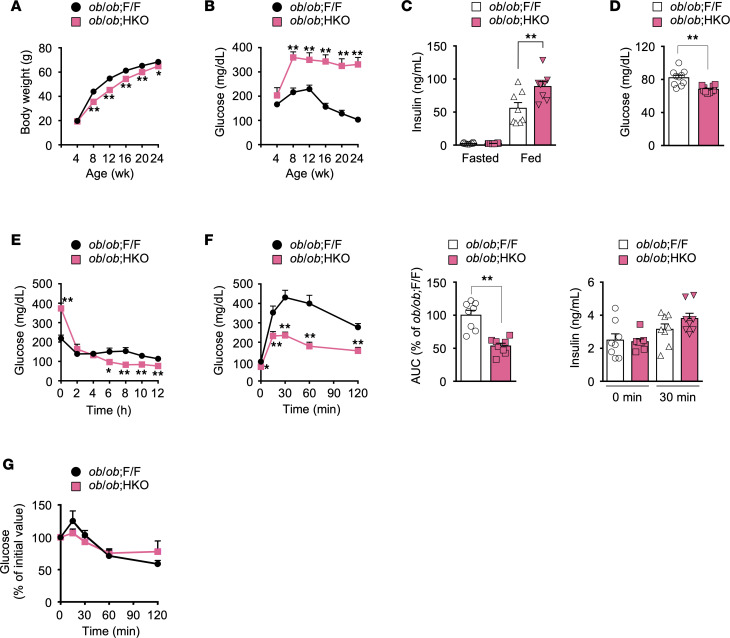
Hepatic FASN deficiency in *ob/ob* mice improves glucose tolerance and confers relative fasting hypoglycemia but exacerbates fed hyperglycemia. (**A** and **B**) BW (**A**) and blood glucose concentration in the fed state (**B**) for *ob/ob* F/F and *ob/ob* HKO mice at the indicated ages (*n* = 10). (**C**) Plasma insulin concentrations of mice in the fasted state at 10 weeks of age (*n* = 8) and in the fed state at 12 weeks of age (*n* = 8). (**D**) Fasting blood glucose concentration in 10- to 12-week-old mice (*n* = 10). (**E**) Blood glucose levels during a 12-hour fasting challenge test in 12-week-old mice (*n* = 8). (**F**) Blood glucose concentrations, AUC, and plasma insulin concentrations during an IPGTT (2 g of glucose per kg of BW) in 10-week-old mice (*n* = 8). (**G**) An ITT (4 U of human regular insulin per kg of BW) for 12-week-old mice (*n* = 5). All data are means + SEM for the indicated numbers (*n*) of mice. *P < 0.05, **P < 0.01 compared with *ob/ob* F/F mice or as indicated (2-tailed Student’s *t* test).

Both *ob/ob* HKO and *ob/ob* F/F mice were indistinguishable with regard to food intake, physical activity, body temperature, and uncoupling protein 1 mRNA abundance in brown adipose tissue (Supplemental Figure 4, A–C). On the other hand, *ob/ob* HKO mice showed a smaller BW gain from 8 to 24 weeks of age (Figure 2A) as well as higher O_2_ consumption and CO_2_ production and a smaller respiratory exchange ratio in the dark phase (Supplemental Figure 4, D–F) compared with *ob/ob* F/F mice. Despite these hallmarks of improved glucose metabolism as well as the amelioration of hepatic steatosis in *ob/ob* HKO mice, these animals manifested exacerbated hyperglycemia and hyperinsulinemia (Figure 2, B and C) without an apparent change in hepatic insulin signaling, as assessed on the basis of Akt phosphorylation at Ser^473^ (Supplemental Figure 4G) in the fed state compared with *ob/ob* F/F mice.

In contrast, *ob/ob* HKO mice had significantly lower blood glucose levels (Figure 2D) accompanied by identical plasma insulin levels (Figure 2C) in the fasted state relative to *ob/ob* F/F mice. The *ob/ob* HKO mice also showed a rapid and more pronounced decline in blood glucose concentration in a 12-hour fasting challenge test (Figure 2E) as well as reduced glucose levels in an IPGTT (Figure 2F) compared with control mice, whereas glucose levels in an ITT were identical for both genotypes (Figure 2G). Plasma insulin secretion at 30 minutes after glucose administration was similarly impaired in both groups of mice (Figure 2F), as previously described for *ob/ob* mice (35). Hepatic insulin signaling — as assessed on the basis of tyrosine phosphorylation of the β subunit of the insulin receptor (IRβ) and Akt phosphorylation at Ser^473^ after insulin administration — was more markedly impaired in *ob/ob* HKO mice than in *ob/ob* F/F mice (Supplemental Figure 4H). These data together thus indicated that hepatic FASN deficiency in *ob/ob* mice improves glucose tolerance and confers relative fasting hypoglycemia but that it exacerbates hyperglycemia in the fed state independently of plasma insulin levels and hepatic insulin signaling.

### Glucose overfeeding exacerbates fed hyperglycemia in ob/ob HKO mice.

**Figure 3:**
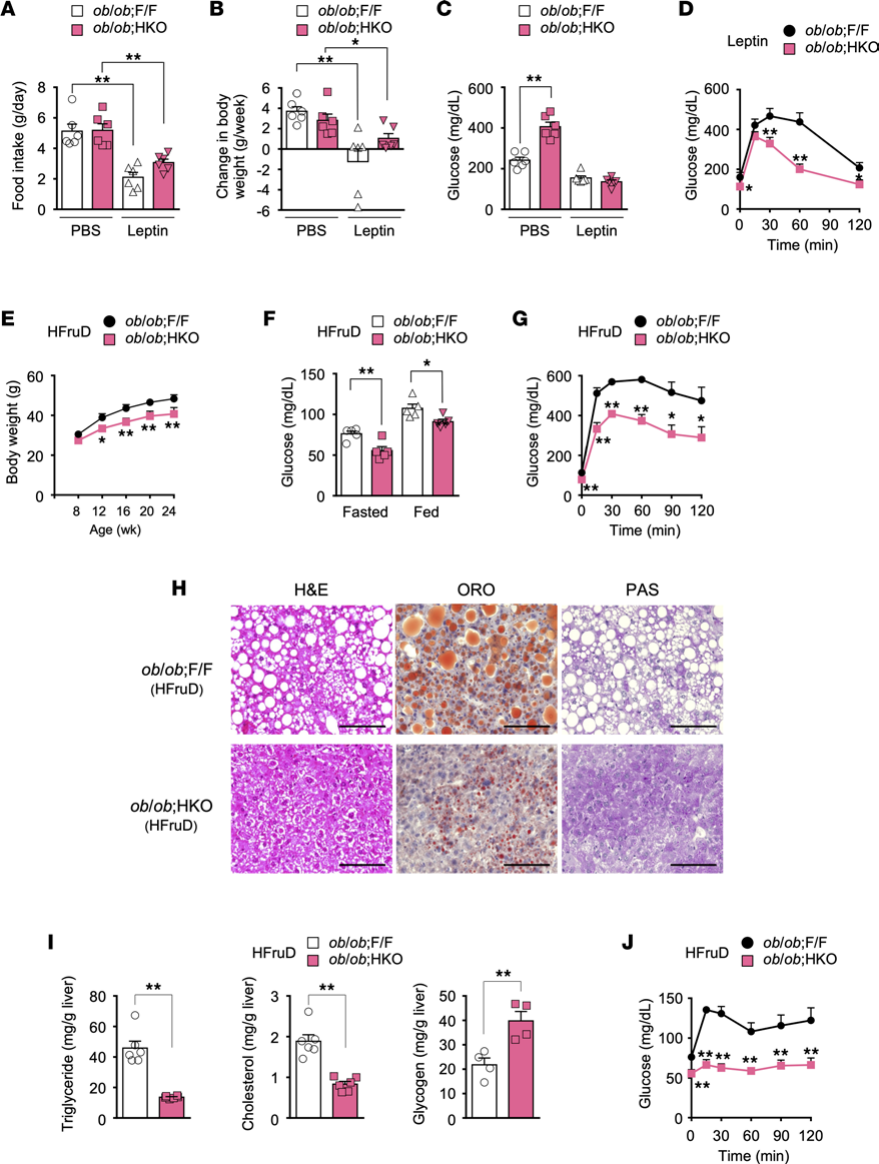
Effects of leptin supplementation and HFruD feeding on the metabolic phenotype of *ob/ob* HKO mice. (**A**–**D**) Food intake (**A**), change in BW (**B**), blood glucose concentration in the fed state (**C**), and the results of an IPGTT (2 g of glucose per kg of BW) (**D**) at 1 week after the onset of leptin or PBS (vehicle) supplementation in 8- to 9-week-old *ob/ob* F/F and *ob/ob* HKO mice (*n* = 6). (**E** and **F**) BW at the indicated ages (*n* = 7) (**E**) and blood glucose concentrations in the fasted and fed states at 24 weeks of age (*n* = 7) (**F**) for HFruD-fed *ob/ob* F/F and *ob/ob* HKO mice. (**G**) An IPGTT (2 g of glucose per kg of BW) in 14-week-old HFruD-fed mice (*n* = 4). (**H**) H&E, Oil Red O, and PAS staining of liver sections from 16-week-old HFruD-fed mice. Images are representative of 4 mice per genotype. Scale bars, 100 μm. (**I**) Hepatic triglyceride (*n* = 6), cholesterol (*n* = 6), and glycogen (*n* = 4) levels in 24-week-old HFruD-fed mice. (**J**) A pyruvate tolerance test for 15-week-old HFruD-fed mice (*n* = 5). All quantitative data are means + SEM for the indicated numbers of mice. *P < 0.05, **P < 0.01 compared with *ob/ob* F/F mice or as indicated (1-way ANOVA followed by Tukey’s multiple comparison test in **A**–**C** or 2-tailed Student’s *t* test in **D**–**G**, **I**, and **J**). HFruD, high-fructose, low-glucose diet; PAS, periodic acid–Schiff.

To clarify how leptin deficiency contributes to the glycemic phenotype of *ob/ob* HKO mice, we performed a leptin supplementation experiment. At 1 week after the onset of leptin administration, both *ob/ob* F/F and *ob/ob* HKO mice showed similar reductions in food intake and in BW gain (Figure 3, A and B), suggestive of effective supplementation. In *ob/ob* HKO mice, leptin administration reversed the exacerbation of fed hyperglycemia, reducing blood glucose levels to values similar to those of *ob/ob* F/F mice (Figure 3C), but it had no effect on the relative fasting hypoglycemia and improved glucose tolerance in these mice (Figure 3D). To address whether the exacerbation of fed hyperglycemia in *ob/ob* HKO mice is induced by glucose overfeeding due to hyperphagia, we analyzed mice fed an HFruD containing 60% fructose and 5% cornstarch by weight. HFruD-fed *ob/ob* HKO mice showed a smaller BW gain relative to *ob/ob* F/F mice (Figure 3E), even though food intake and the plasma fructose concentration were similar for both genotypes (Supplemental Figure 5, A and B). Unlike those fed an NCD, *ob/ob* HKO mice fed the HFruD did not show exacerbated hyperglycemia, but rather manifested hypoglycemia, in the fed state compared with *ob/ob* F/F mice (Figure 3F), indicative of a causal role of glucose overfeeding in the exacerbation of hyperglycemia. With this exception, HFruD-fed *ob/ob* HKO mice showed a similar phenotype to NCD-fed *ob/ob* HKO mice that was characterized by relative fasting hypoglycemia, improved glucose tolerance, and ameliorated hepatic steatosis (Figure 3, F–I), albeit with an unchanged plasma insulin level in the fed state (Supplemental Figure 5C).

HFruD-fed *ob/ob* HKO mice also manifested a higher hepatic glycogen level in the fed state, as assessed both by PAS staining of liver sections and by a colorimetric assay (Figure 3, H and I, and Supplemental Figure 5D), compared with corresponding *ob/ob* F/F mice. However, hepatic glycogenesis signaling in the fed state, assessed on the basis of phosphorylation of glycogen synthase kinase–3α/β at Ser^21/9^ and of glycogen synthase (GS) at Ser^641^, was not enhanced in HFruD-fed *ob*/*ob* HKO mice (Supplemental Figure 5E). In addition, the activity of hepatic glycogen phosphorylase was not decreased but was increased in HFruD-fed *ob*/*ob* HKO mice in the fasted state and was similar between the 2 genotypes in the fed state (Supplemental Figure 5F). These data suggested that hepatic glycogen accumulation in HFruD-fed *ob*/*ob* HKO mice was not associated with changes in signaling that regulates glycogen synthesis and breakdown. Hepatic glycogen accumulation in the presence of hypoglycemia is reminiscent of glycogen storage disease type Ia (also known as von Gierke disease), which is caused by genetic deficiency of glucose-6-phosphatase (G6Pase) activity and is characterized by impaired gluconeogenesis and glycogenolysis in the liver (36). Given that HFruD-fed mice have a restricted oral glucose supply and that fructose serves as a gluconeogenic precursor, hepatic gluconeogenesis may be a key determinant of blood glucose levels in these animals. To examine the role of hepatic gluconeogenesis in the hypoglycemic phenotype of HFruD-fed *ob/ob* HKO mice, we performed a pyruvate tolerance test. Glucose levels after pyruvate administration were markedly lower in *ob/ob* HKO mice compared with *ob/ob* F/F mice (Figure 3J), suggestive of the suppression of hepatic gluconeogenesis. Such suppression might thus account, at least in part, for the hypoglycemic phenotype of HFruD-fed *ob/ob* HKO mice. Together, these data suggested that the exacerbated hyperglycemia of NCD-fed *ob/ob* HKO mice in the fed state is attributable to glucose overfeeding associated with leptin deficiency, whereas the hypoglycemic phenotype of HFruD-fed *ob/ob* HKO mice may be associated with reduced hepatic gluconeogenesis but not with leptin deficiency.

### Hepatic FASN deficiency in ob/ob mice suppresses gluconeogenesis and enhances glucose uptake in the liver.

**Figure 4:**
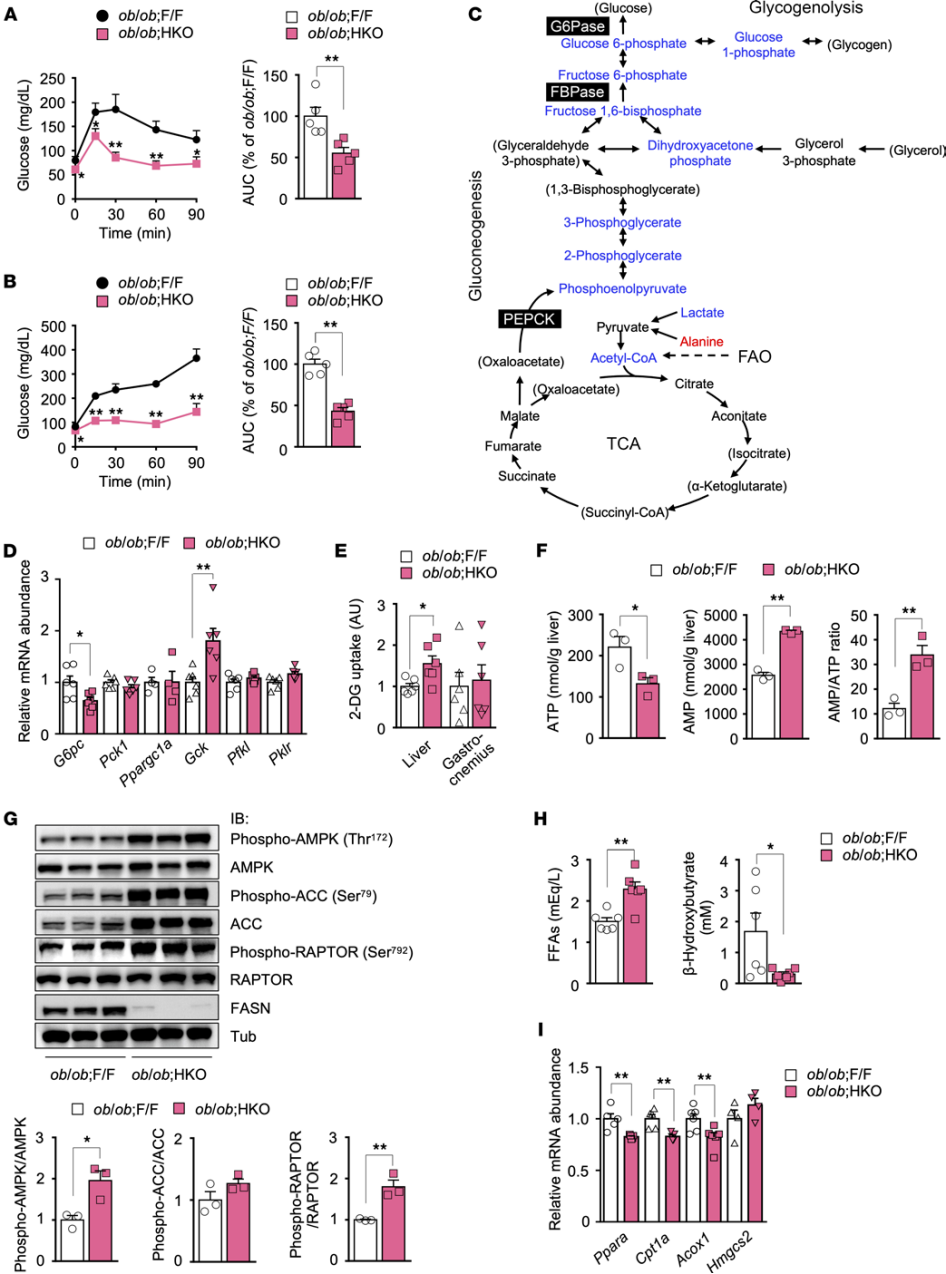
Hepatic FASN deficiency in *ob/ob* mice suppresses gluconeogenesis in association with inhibition of PPARα and FAO and activation of AMPK. (**A** and **B**) Pyruvate (**A**) and glycerol (**B**) tolerance tests in 10-week-old *ob/ob* F/F and *ob/ob* HKO mice (*n* = 5). (**C**) Metabolites in the liver of fasted 10-week-old mice (*n* = 4) were measured by mass spectrometry. The results are depicted as a pathway activity map, with red and blue indicating metabolites whose abundance was increased or decreased, respectively, in *ob/ob* HKO mice compared with *ob/ob* F/F mice. Metabolites in parentheses were not detected. Quantitative data are provided in Supplemental Table 1. FBPase, fructose-1,6-bisphosphatase; PEPCK, phosphoenolpyruvate carboxykinase. (**D**) RT-qPCR analysis of gene expression related to gluconeogenesis or glycolysis in the liver of fasted 10-week-old mice (*n* = 4 to 6). (**E**) 2-DG uptake in the liver and gastrocnemius muscle of 10- to 12-week-old mice (*n* = 6). (**F**) Hepatic ATP and AMP levels as well as the AMP/ATP ratio in fasted 10-week-old mice (*n* = 3). (**G**) Immunoblot analysis of phosphorylated and total forms of AMPKα subunits, ACC, and RAPTOR in the liver of fasted 10-week-old mice (*n* = 3). (**H**) Plasma FFA and β-hydroxybutyrate levels in fasted 10-week-old mice (*n* = 6). (**I**) RT-qPCR analysis of the expression of PPARα target genes related to FAO or ketogenesis in the liver of fasted 10-week-old mice (*n* = 4 to 6). All quantitative data are means + SEM for the indicated numbers of mice. *P < 0.05, **P < 0.01 compared with *ob/ob* F/F mice or as indicated (2-tailed Student’s *t* test). *Gck*, glucokinase; *Pfkl*, liver-type phosphofructokinase.

Given that relative hypoglycemia and improved glucose tolerance in HFruD-fed *ob/ob* HKO mice were associated with reduced hepatic gluconeogenesis (Figure 3, F, G, and J), we examined the role of hepatic gluconeogenesis in the hypoglycemic phenotype of NCD-fed *ob/ob* HKO mice. Blood glucose levels during pyruvate tolerance and glycerol tolerance tests were significantly lower in *ob/ob* HKO mice (Figure 4, A and B) than in *ob/ob* F/F mice. Metabolomic profiling of the liver of fasted animals revealed that the levels of gluconeogenic and glycogenolytic intermediates as well as of acetyl-CoA and lactate were lower, whereas that of alanine was higher, in *ob/ob* HKO mice compared with *ob/ob* F/F mice (Figure 4C and Supplemental Table 1). These findings were compatible with suppression of hepatic gluconeogenesis in NCD-fed *ob/ob* HKO mice. Analysis of hepatic gene expression in the fasted state revealed that expression of the gene for the catalytic subunit of G6Pase (*G6pc*) was decreased by 36%, whereas that of the genes for phosphoenolpyruvate carboxykinase (*Pck1*) and PPARγ coactivator 1α (*Ppargc1a*) was not altered, in *ob/ob* HKO mice compared with *ob/ob* F/F mice, consistent with a reduced level of gluconeogenesis in the former mice (Figure 4D). Unexpectedly, expression of the gene *Gck*, whose upregulation in the liver has been shown to promote hepatic glucose uptake (HGU) (37, 38), was almost doubled in the liver of *ob/ob* HKO mice despite unaltered expression of genes for other key glycolytic enzymes such as *Pfkl* and *Pklr* (Figure 4D). Given that these reciprocal changes in *G6pc* and *Gck* expression during fasting would be expected to promote HGU after a glucose load, we evaluated HGU with 2-deoxyglucose (2-DG) as a tracer. 2-DG uptake was increased by 50% in the liver but was unaffected in gastrocnemius muscle of *ob/ob* HKO mice compared with *ob/ob* F/F mice (Figure 4E), suggestive of enhanced HGU in *ob/ob* HKO mice. Collectively, these data indicated that hepatic FASN deficiency in *ob/ob* mice suppresses gluconeogenesis and may promote HGU; the suppression of gluconeogenesis may contribute, at least in part, to fasting hypoglycemia and improved glucose tolerance, while the promotion of HGU may be associated with improved glucose tolerance.

### Hepatic FASN deficiency in ob/ob mice impairs FAO and activates AMPK.

Gluconeogenesis requires acetyl-CoA and ATP (39). In the fasted liver, ATP is produced mainly from catabolism of acetyl-CoA, which is provided predominantly by mitochondrial FAO. Given that our metabolite analysis revealed that the fasting levels of acetyl-CoA and gluconeogenic intermediates in the liver were reduced for *ob/ob* HKO mice compared with *ob/ob* F/F mice (Figure 4C), we investigated adenine nucleotide levels and FAO in the liver. The ATP level was lower and that of AMP and the AMP/ATP ratio were higher in the liver of fasted *ob/ob* HKO mice compared with the control liver (Figure 4F). Consistent with this increase in the AMP/ATP ratio, AMPK was markedly activated in the *ob/ob* HKO mouse liver, as reflected by increased phosphorylation of the α subunits of AMPK at Thr^172^ and of the AMPK substrate RAPTOR at Ser^792^ (Figure 4G). These findings thus suggested that the reduction in hepatic ATP and acetyl-CoA levels (39), the increase in hepatic AMP abundance (40, 41), and the activation of AMPK (42) may cooperatively suppress gluconeogenesis in *ob/ob* HKO mice.

In response to energy deprivation, AMPK promotes FAO to replenish ATP stores by attenuating malonyl-CoA production through inhibitory phosphorylation of ACC (43). However, fasting plasma levels of FFAs and β-hydroxybutyrate, a ketone body synthesized from FAO-derived acetyl-CoA and a surrogate biomarker for hepatic FAO, were higher and lower, respectively, in *ob/ob* HKO mice than in *ob/ob* F/F mice (Figure 4H), suggestive of impaired hepatic FAO that might give rise to suppression of gluconeogenesis in *ob/ob* HKO mice. In the liver of fasted animals, activation of PPARα (encoded by *Ppara*) promotes FAO and ketogenesis through the induction of FAO-related genes, such as *Cpt1a* (encoding carnitine palmitoyltransferase 1A) and *Acox1* (encoding acyl-CoA oxidase 1), and of ketogenesis-related genes, such as *Hmgcs2* (encoding HMG-CoA synthase 2) (44). Given that FASN activity is necessary for synthesis of an endogenous ligand for PPARα in the liver of nonobese, ZFD-fed mice (45), we tested whether hepatic FASN deficiency in *ob/ob* mice might also affect PPARα activity. Hepatic expression of *Ppara* and of the PPARα target genes *Cpt1a* and *Acox1* was significantly decreased in fasted *ob/ob* HKO mice compared with *ob/ob* F/F control mice (Figure 4I), suggestive of impaired PPARα activity. Together, our observations thus suggested that hepatic FASN deficiency in *ob/ob* mice reduces PPARα activity and may thereby suppress FAO, leading to reduced ATP and acetyl-CoA levels and an increased AMP abundance. These changes in metabolite levels may cooperatively suppress hepatic gluconeogenesis, resulting in relative fasting hypoglycemia and improved glucose tolerance.

### Hepatic glycogen accumulation and suppression of glycolysis coordinately exacerbate fed hyperglycemia in ob/ob HKO mice.

**Figure 5:**
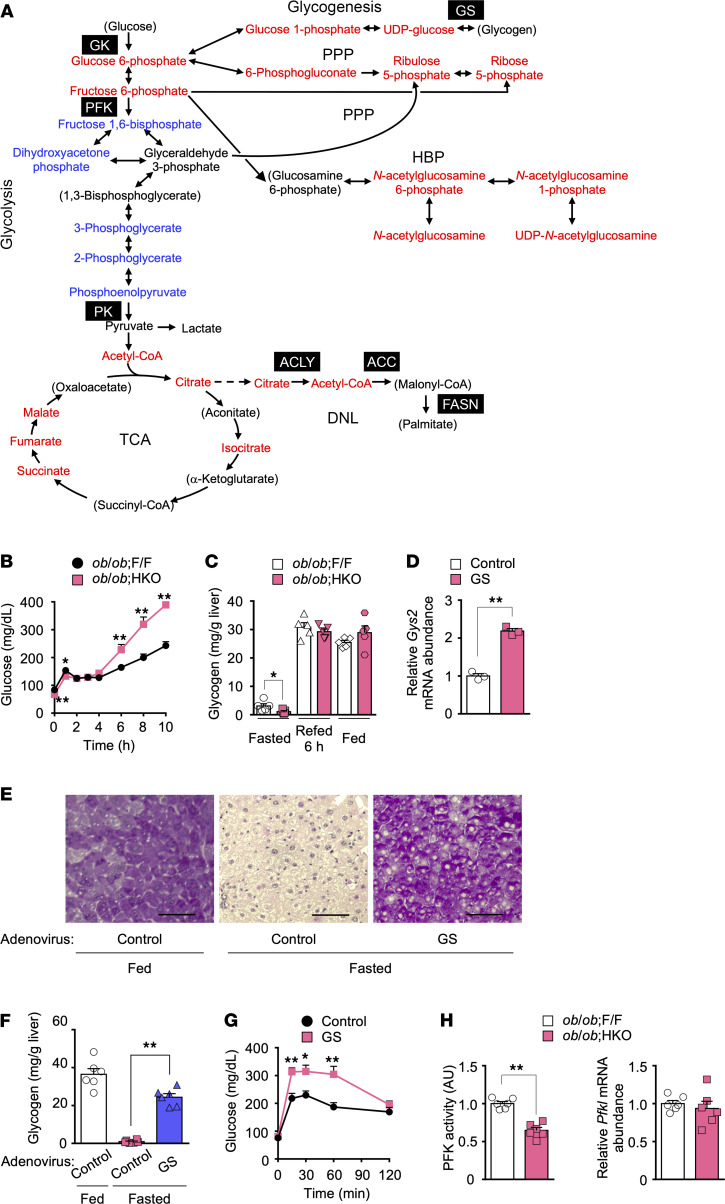
Hepatic glycogen accumulation and suppression of glycolysis coordinately exacerbate fed hyperglycemia in *ob*/*ob* HKO mice. (**A**) Metabolomic profiling of the liver of NCD-fed *ob/ob* F/F and *ob/ob* HKO mice in the fed state at 10 weeks of age (*n* = 3 to 6). Liver metabolites were measured by mass spectrometry. Results are depicted as a pathway activity map; red and blue indicate metabolites with an increased or decreased abundance, respectively, in *ob/ob* HKO mice compared with *ob/ob* F/F mice. Metabolites in parentheses were not detected. Quantitative data are provided in Supplemental Table 2. GK, glucokinase; PFK, phosphofructokinase; PPP, pentose phosphate pathway; HBP, hexosamine biosynthesis pathway; PK, pyruvate kinase. (**B**) Blood glucose concentrations at the indicated times during refeeding after food deprivation for 16 hours in 10- to 12-week-old *ob/ob* F/F and *ob/ob* HKO mice (*n* = 10). (**C**) Hepatic glycogen content in fasted (16 hours), refed (6 hours), and fed states for 10- to 12-week-old *ob/ob* F/F and *ob/ob* HKO mice (*n* = 5). (**D**) RT-qPCR analysis of *Gys2* mRNA in the liver of fasted 10-week-old *ob/ob* HKO mice injected with an adenovirus encoding GS or a control virus (*n* = 3). (**E**) PAS staining of liver sections from 10-week-old *ob/ob* HKO mice in the fed or overnight-fasted state after injection with control or GS adenoviruses. Images are representative of 4 mice per condition. Scale bars, 200 μm. (**F**) Hepatic glycogen content in *ob/ob* HKO mice (*n* = 6) as in **E**. (**G**) An IPGTT (2 g of glucose per kg of BW) in *ob/ob* HKO mice injected with control or GS adenoviruses (*n* = 6). (**H**) Activity and mRNA abundance for liver-type PFK (encoded by *Pfkl*) in the liver of 10-week-old *ob/ob* F/F and *ob/ob* HKO mice in the fed state (*n* = 6). All quantitative data are means + SEM for the indicated numbers of mice. *P < 0.05, **P < 0.01 compared with *ob/ob* F/F mice or the control adenovirus, or as indicated (2-tailed Student’s *t* test in **B**–**D**, **G**, and **H** or 1-way ANOVA followed by Tukey’s multiple-comparison test in **F**).

We next sought to uncover the mechanism responsible for exacerbation of fed hyperglycemia in *ob/ob* HKO mice, which was induced by overfeeding with glucose but not with fructose (Figure 3F). Metabolomic profiling of the liver in the fed state revealed an increase in the abundance of DNL intermediates upstream of FASN (acetyl-CoA and citrate) and in that of TCA cycle intermediates in *ob/ob* HKO mice (Figure 5A and Supplemental Table 2), consistent with FASN deficiency. It also revealed an increase in the amounts of upstream metabolites of PFK in glycolysis, glycogenesis, the PPP, and the HBP as well as a decrease in those of downstream metabolites of PFK in glycolysis in *ob/ob* HKO mice compared with *ob/ob* F/F mice (Figure 5A), consistent with impairment of glycolysis at the PFK level. In this setting, glucose might be expected to be metabolized via glycogenesis, the PPP, and the HBP.

Given that glycogen serves as the largest glucose reservoir (46), we tested whether hepatic glycogen accumulation may affect blood glucose levels in *ob/ob* HKO mice. In a fasting and refeeding experiment, *ob/ob* HKO mice manifested higher blood glucose levels compared with *ob/ob* F/F mice at ≥6 hours after the onset of refeeding (Figure 5B). Unlike in HFruD-fed animals (Figure 3I), hepatic glycogen levels at 6 hours after the onset of refeeding as well as in the fed state were identical in both groups of mice (Figure 5C). These results suggested that glycogenesis capacity is not altered but that glycogen accumulation is correlated with exacerbated hyperglycemia in *ob/ob* HKO mice in the fed and postprandial states. To assess directly the causal link between hepatic glycogen accumulation and the exacerbation of hyperglycemia, we performed glycogen replenishment analysis for the liver of fasted *ob/ob* HKO mice infected with an adenovirus vector encoding GS (encoded by *Gys2*) (47). Overexpression of GS (Figure 5D) increased the hepatic glycogen content (as assessed by PAS staining and a colorimetric assay) to a level similar to that apparent in fed *ob/ob* HKO mice injected with a control adenovirus (Figure 5, E and F). This replenishment of hepatic glycogen was associated with an increase in blood glucose levels during an IPGTT (Figure 5G). These findings thus suggested that hepatic glycogen accumulation drives the exacerbation of hyperglycemia in *ob/ob* HKO mice.

Given that our metabolomic profiling suggested that FASN deficiency in *ob/ob* mice may result in inhibition of PFK (Figure 5A), which is allosterically inhibited by citrate (48), we evaluated the expression and activity of this enzyme. Consistent with the observed increase in the hepatic citrate level (Figure 5A), hepatic PFK activity was reduced by 35% in *ob/ob* HKO mice compared with *ob/ob* F/F mice, despite the absence of a difference in *Pfkl* mRNA levels (Figure 5H). Together, these observations suggested that, under fed and late postprandial conditions, hepatic FASN deficiency may inhibit glycolysis through citrate-mediated suppression of PFK activity in *ob/ob* mice. This inhibition and hepatic glycogen accumulation may cooperatively restrain glucose utilization and uptake, thereby exacerbating fed hyperglycemia.

### Hepatic FASN deficiency in Mc4r-KO mice ameliorates NAFLD and diabetes.

**Figure 6:**
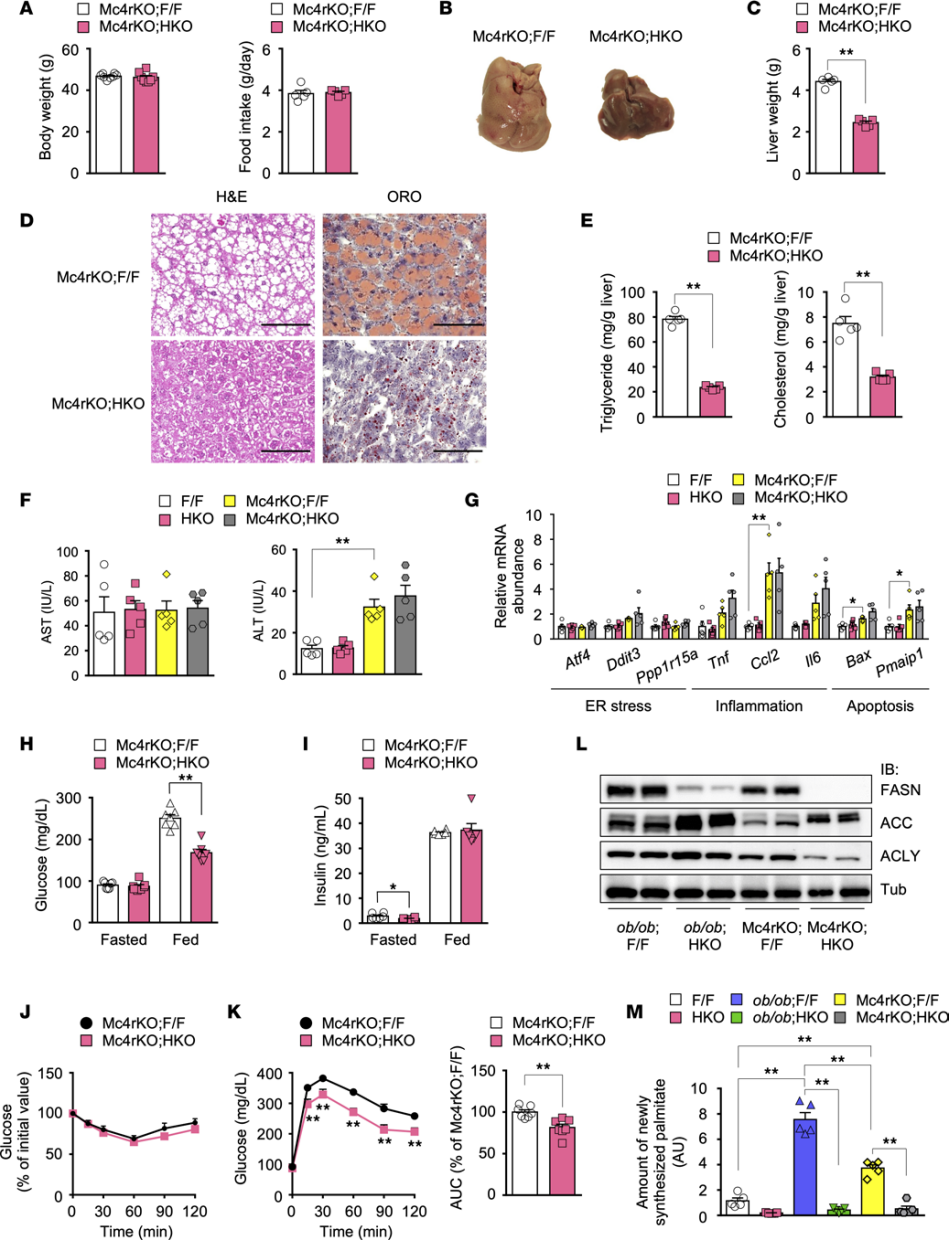
Hepatic FASN deficiency in Mc4r-KO mice ameliorates NAFLD and diabetes. (**A**–**E**) BW (*n* = 8) and 24-hour food intake (*n* = 5) (**A**), macroscopic appearance of the liver (**B**), liver weight (*n* = 5) (**C**), H&E and Oil Red O staining of liver sections (**D**), and hepatic triglyceride and cholesterol levels in the fed state (*n* = 5) (**E**) for 16- to 19-week-old Mc4r-KO F/F and Mc4r-KO HKO mice maintained on an NCD. All images are representative of 5 mice per group. Scale bars, 100 μm (**D**). (**F** and **G**) Plasma transaminase levels (*n* = 5) (**F**) as well as RT-qPCR analysis of hepatic gene expression related to ER stress, inflammation, or apoptosis (*n* = 5) (**G**) for 16- to 20-week-old fed mice of the indicated genotypes maintained on an NCD. (**H** and **I**) Blood glucose (*n* = 7) (**H**) and plasma insulin (*n* = 6) (**I**) levels in the fasted and fed states for mice at 16 to 18 weeks of age. (**J** and **K**) An ITT (2 U of human regular insulin per kg of BW) (*n* = 8) (**J**) and IPGTT (1.5 g of glucose per kg of BW) (*n* = 7) (**K**) for 16- to 19-week-old mice. (**L**) Immunoblot analysis of lipogenic enzymes in the fed liver of 10-week-old *ob/ob* F/F and *ob/ob* HKO mice as well as 16- to 18-week-old Mc4r-KO F/F and Mc4r-KO HKO mice maintained on an NCD. (**M**) Hepatic abundance of newly synthesized palmitate in the fasted state (6 hours) for 10-week-old F/F, HKO, *ob/ob* F/F, and *ob/ob* HKO mice and for 16- to 18-week-old Mc4r-KO F/F and Mc4r-KO HKO mice maintained on an NCD (*n* = 5). All quantitative data are means + SEM for the indicated numbers of mice. *P < 0.05, **P < 0.01 compared with Mc4r-KO F/F mice or as indicated (2-tailed Student’s *t* test [**C**, **E**, **H**, **I**, and **K**] or 1-way ANOVA followed by Bonferroni’s multiple-comparison test [**F**, **G**, and **M**]).

Our results suggested that, in NCD-fed *ob/ob* HKO mice, the exacerbated hyperglycemia in the fed state is attributable to leptin deficiency–dependent glucose overfeeding, whereas the hypoglycemic phenotype is independent of leptin deficiency (Figure 3). To clarify the effects of hepatic FASN deficiency on NAFLD and diabetes in obese mice in the setting of NCD overfeeding without leptin deficiency, we next studied Mc4r-KO mice as an alternative mouse model of hyperphagic obesity associated with hepatic FASN upregulation and hyperleptinemia (49–51). Mc4r-KO F/F mice at 16 to 19 weeks of age (Figure 6A) had a BW similar to that of 10- to 12-week-old *ob/ob* F/F mice (Figure 2A) and manifested overt hepatic steatosis accompanied by liver dysfunction — as assessed on the basis of the gross appearance and weight of the liver, Oil Red O staining of liver sections, hepatic lipid content, plasma transaminase levels, and hepatic expression of genes related to ER stress, inflammation, and apoptosis—compared with F/F mice (Figure 6, B–G). They also showed hyperglycemia and hyperinsulinemia in the fed state (Figure 6, H and I) as well as insulin resistance and glucose intolerance as assessed by an ITT and IPGTT, respectively (Figure 6, J and K). Compared with Mc4r-KO F/F mice, Mc4r-KO HKO mice showed a similar BW and food intake (Figure 6A) as well as attenuated hepatic steatosis (Figure 6, B–E) and glucose intolerance (Figure 6K). Unexpectedly, unlike *ob/ob* HKO mice, Mc4r-KO HKO mice manifested significantly attenuated hyperglycemia in the fed state (Figure 6H) without exacerbation of liver dysfunction and enhanced expression of related genes in the liver (Figure 6, F and G).

Compared with *ob/ob* F/F mice, Mc4r-KO F/F mice had a 49% lower hepatic triglyceride content (Figure 1E and Figure 6E) and approximately 52% lower plasma transaminase levels (Figure 1F and Figure 6F), indicative of less pronounced NAFLD. These differences were associated with a 40% lower food intake (Figure 6A and Supplemental Figure 4A), 35% lower level of hyperinsulinemia in the fed state (Figure 2C and Figure 6I), and lower protein expression of hepatic lipogenic enzymes (Figure 6L and Supplemental Figure 6A) in Mc4r-KO F/F mice. Hepatic expression of target genes of SREBP1c (*Acly*, *Pnpla3*), ChREBP (*Pklr*, *Aldob*), or both of these transcription factors (*Acc1*, *Fasn*, *Scd1*, *Elovl6*) was also lower in Mc4r-KO F/F mice compared with *ob/ob* F/F mice (Supplemental Figure 6B), suggesting that DNL may be increased to a lesser extent in Mc4r-KO F/F mice. In addition, hepatic FASN deficiency ameliorated hepatic steatosis to a lesser extent in Mc4r-KO mice than in *ob/ob* mice, as assessed on the basis of hepatic triglyceride content (70% versus 96% inhibition) (Figure 1E and Figure 6E). Consistent with these findings, the activity of DNL in the liver, assessed on the basis of the amount of newly synthesized palmitate, was increased to a lesser extent in Mc4r-KO F/F mice relative to *ob*/*ob* F/F mice (Figure 6M). Collectively, these data indicated that, in Mc4r-KO mice — a model of hyperphagic obesity without leptin deficiency in which hepatic DNL is increased to a lesser extent than in *ob/ob* mice — hepatic FASN deficiency ameliorated both NAFLD and diabetes.

### Hepatic FASN deficiency in Mc4r-KO mice suppresses FAO and gluconeogenesis in association with augmentation of insulin signaling.

**Figure 7:**
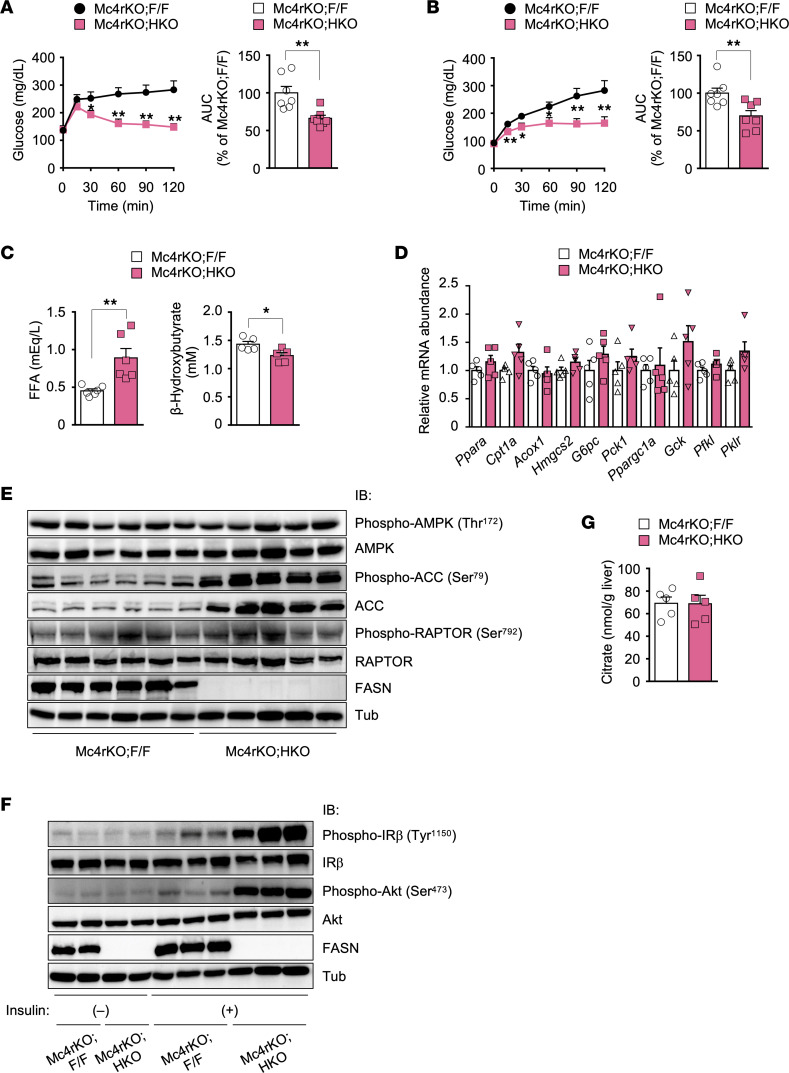
Hepatic FASN deficiency in Mc4r-KO mice improves glucose metabolism by inhibiting gluconeogenesis and augmenting insulin signaling. (**A** and **B**) Pyruvate (**A**) and glycerol (**B**) tolerance tests for 20-week-old Mc4r-KO F/F and Mc4r-KO HKO mice (*n* = 7). (**C**) Plasma FFA and β-hydroxybutyrate levels in fasted 16- to 20-week-old mice (*n* = 6). (**D**) RT-qPCR analysis of the expression of PPARα target genes related to FAO or ketogenesis as well as of genes related to gluconeogenesis or glycolysis in the liver of fasted 16- to 20-week-old mice (*n* = 6). (**E**) Immunoblot analysis of phosphorylated and total forms of AMPKα subunits, ACC, and RAPTOR in the liver of fasted 16- to 20-week-old mice (*n* = 5 or 6). (**F**) Effects of insulin on IRβ and Akt phosphorylation in the liver of 16- to 20-week-old mice. Mice deprived of food overnight were injected intravenously with insulin (5 U/kg) or PBS (–), 2 minutes after which the liver was isolated, lysed, and subjected to immunoblot analysis. Each lane corresponds to 1 mouse, and the blots are representative of 2 independent experiments. (**G**) Hepatic citrate levels in 20-week-old Mc4r-KO F/F and Mc4r-KO HKO mice in the fed state (*n* = 5). All quantitative data are means + SEM for the indicated numbers of mice. *P < 0.05, **P < 0.01 compared with Mc4r-KO F/F mice or as indicated (2-tailed Student’s *t* test).

Given that the reduced glycemia and improved glucose tolerance in *ob*/*ob* HKO mice fed an HFruD (Figure 3, F and G) or NCD (Figure 2, D and F, and Figure 4, A and B) compared with respective control mice were associated with attenuated hepatic gluconeogenesis, we examined the role of hepatic gluconeogenesis in Mc4r-KO HKO mice. Blood glucose levels during pyruvate tolerance and glycerol tolerance tests were significantly lower in Mc4r-KO HKO mice than in Mc4r-KO F/F mice (Figure 7, A and B), but these differences were smaller than those between *ob*/*ob* HKO and control mice (Figure 4, A and B), suggesting that hepatic FASN deficiency suppresses gluconeogenesis in Mc4r-KO mice but to a lesser extent than it does in *ob*/*ob* mice. This suppression was associated with higher FFA and lower β-hydroxybutyrate levels in plasma in fasted Mc4r-KO HKO mice than in control mice (Figure 7C), but again these differences were smaller than those apparent for *ob*/*ob* HKO and control mice (Figure 4H), suggesting that hepatic FASN deficiency suppresses FAO to a lesser extent in Mc4r-KO mice than in *ob*/*ob* mice. Unexpectedly, Mc4r-KO HKO mice, unlike *ob*/*ob* HKO mice, manifested neither a reduced level of expression for genes related to FAO, gluconeogenesis, or glycolysis (Figure 7D) nor enhanced AMPK activation, as assessed by phosphorylation of AMPK and its substrates (Figure 7E), in the liver, but they showed markedly augmented hepatic insulin signaling (Figure 7F) compared with Mc4r-KO F/F mice. Consistent with the lack of exacerbation of fed hyperglycemia in Mc4r-KO HKO mice, the hepatic citrate level in these mice was identical to that in Mc4r-KO F/F mice (Figure 7G). Collectively, these findings suggested that hepatic FASN deficiency in Mc4r-KO mice suppresses FAO and gluconeogenesis in association with augmentation of insulin signaling, which together may ameliorate diabetes. However, the mechanisms of such suppression appear to differ between the 2 mouse models of genetic obesity, with hepatic FASN deficiency attenuating PPARα activity and activating AMPK in *ob*/*ob* mice but not in Mc4r-KO mice.

## Discussion

The goal of this study was to examine the therapeutic potential of targeting hepatic FASN for NAFLD and diabetes with the use of HKO mice with various types of obesity. We have shown that hepatic FASN deficiency differentially affects NAFLD and diabetes in a manner dependent on the genetic and dietary background of obesity. Such deficiency thus ameliorated NAFLD and diabetes in Mc4r-KO mice but not in HFD-fed mice. Moreover, whereas hepatic FASN ablation ameliorated hepatic steatosis and improved glucose tolerance in NCD-fed *ob*/*ob* mice to a greater extent than in Mc4r-KO mice, it also exacerbated both hyperglycemia in the fed state and liver dysfunction.

Hepatic FASN deficiency was previously shown to exacerbate hepatic steatosis and liver dysfunction as well as to induce relative hypoglycemia and improve glucose tolerance in nonobese mice fed a ZFD but not in those fed a NCD (30). These changes were associated with reduced hepatic FAO and were reversed by administration of a PPARα agonist or by feeding an NCD containing 5% fat (30). A subsequent study found that hepatic FASN deficiency impairs the production of an endogenous PPARα ligand, resulting in downregulation of the expression of PPARα target genes related to FAO (45). Unlike in ZFD-fed mice, we found that hepatic FASN ablation ameliorated steatosis in *ob*/*ob* mice and, to a lesser extent, in Mc4r-KO mice. Leptin receptor signaling and Mc4r signaling exert antiobesity effects mainly through suppression of food intake and increased energy consumption (50, 52). Our comparison of BW-matched animals revealed that hepatic DNL, FAO, and steatosis as well as hyperphagia, hyperinsulinemia, and hepatic upregulation of lipogenic enzymes were more pronounced in *ob*/*ob* mice compared with Mc4r-KO mice. Enhanced DNL is a major contributor to hepatic steatosis both in these 2 mouse models of genetic obesity and in obese patients with NAFLD (8), suggesting that inhibition of DNL by hepatic FASN deficiency may ameliorate hepatic steatosis in a manner dependent on the extent of the enhancement of DNL. Despite the marked amelioration of hepatic steatosis, hepatic FASN deficiency in *ob*/*ob* mice, but not that in Mc4r-KO mice, exacerbated liver dysfunction, with this effect being associated with hepatic upregulation of gene expression related to ER stress, inflammation, and apoptosis, implicating these processes in the exacerbation of liver dysfunction. Whereas the mechanisms underlying the activation of these pathways remain to be elucidated, the more pronounced suppression of hepatic DNL or FAO, fed hyperglycemia, impaired hepatic insulin signaling, and metabolic stress are potential contributing factors.

**Figure 8:**
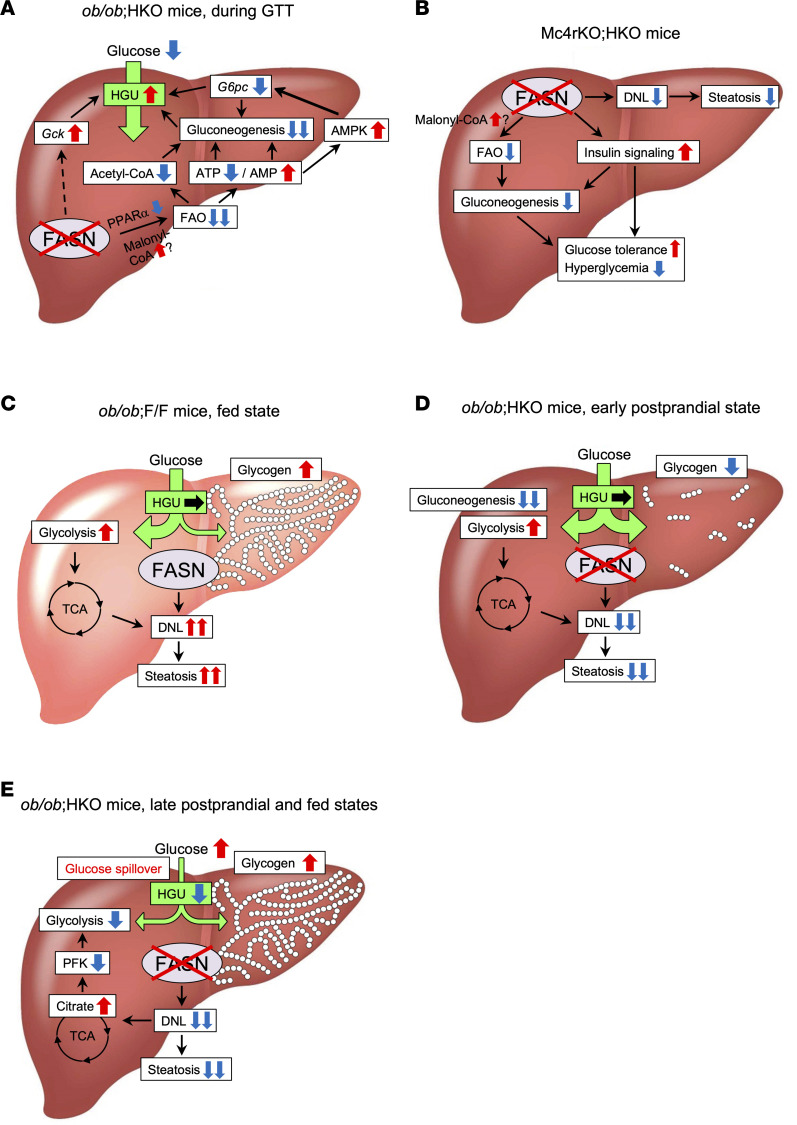
Proposed mechanisms by which hepatic FASN deficiency in NCD-fed *ob*/*ob* or Mc4r-KO mice affects NAFLD and diabetes. (**A**) In the liver of fasted *ob/ob* HKO mice, FAO is impaired, which results in suppression of gluconeogenesis via AMPK-dependent and -independent mechanisms and thereby leads to fasting hypoglycemia. *Gck* expression is also upregulated by an unknown mechanism. Suppression of gluconeogenesis and upregulation of GK together promote HGU during an IPGTT, resulting in improved glucose tolerance. (**B**) In the liver of Mc4r-KO HKO mice, suppression of DNL alleviates hepatic steatosis. Gluconeogenesis is also suppressed as a result of inhibition of FAO and augmentation of insulin signaling. This suppression of gluconeogenesis and enhanced insulin signaling cooperatively improve glucose metabolism. (**C**) In the liver of fed *ob/ob* F/F mice, dietary glucose is metabolized predominantly via glycolysis, the TCA cycle, and DNL as a result of sufficient glycogen accumulation. (**D**) In the liver of *ob/ob* HKO mice, DNL and gluconeogenesis are suppressed, resulting in alleviation of hepatic steatosis and promotion of glucose uptake. In the early postprandial state, dietary glucose is therefore metabolized predominantly through glycolysis, the TCA cycle, and glycogenesis, resulting in maintenance of blood glucose levels similar to those of *ob/ob* F/F mice. (**E**) In the liver of *ob/ob* HKO mice in the late postprandial and fed states, glycolysis is inhibited through citrate-mediated suppression of PFK activity. This inhibition of glycolysis and hepatic glycogen accumulation cooperatively restrain glucose utilization and uptake, resulting in glucose spillover and consequent exacerbation of fed hyperglycemia.

With regard to glucose metabolism, hepatic FASN deficiency in *ob*/*ob* mice lowered fasting glycemia and improved glucose tolerance, at least in part through suppression of hepatic gluconeogenesis independently of insulin signaling. Metabolomic and biochemical analyses of the liver of fasted *ob/ob* mice revealed that hepatic FASN ablation increased the abundance of AMP and reduced that of ATP, increased the AMP/ATP ratio, and reduced the amount of acetyl-CoA, with these effects being mediated, at least in part, through suppression of FAO in association with PPARα inhibition (Figure 8A). An increase in AMP abundance may result in the allosteric inhibition of both adenylate cyclase (40), a key enzyme that generates cAMP in response to glucagon and thereby activates the gluconeogenic program, and fructose 1,6-bisphosphatase (41), a rate-limiting enzyme for gluconeogenesis. An increase in the AMP/ATP ratio results in the activation of AMPK, which may suppress the glucagon-dependent transcription of gluconeogenic genes such as *G6pc* (42), whereas a drop in ATP and acetyl-CoA levels results in suppression of ATP-dependent processes of gluconeogenesis and pyruvate carboxylase flux, respectively (39, 53). These changes could cooperatively suppress hepatic gluconeogenesis (Figure 8A). In addition, the upregulation of *Gck* expression induced by hepatic FASN ablation in *ob/ob* mice, the mechanism of which remains unknown, might also promote HGU and lower blood glucose levels after glucose loading (Figure 8A). Hepatic FASN deficiency in Mc4r-KO mice suppressed FAO in association with enhancement of hepatic insulin signaling, both of which might suppress gluconeogenesis; these effects were not accompanied by PPARα inhibition or AMPK activation, however, and may have contributed to the amelioration of fed hyperglycemia and improvement of glucose tolerance (Figure 8B).

In ZFD-fed HKO mice, suppression of FAO was found to occur as a result of impaired production of an endogenous PPARα ligand and was reversed by NCD feeding, suggesting that dietary fat intake restored the production of this ligand by bypassing the FASN-dependent process (30, 45). However, in NCD-fed *ob*/*ob* HKO and Mc4r-KO HKO mice, suppression of FAO was maintained despite dietary fat intake, which may be explained by impairment of biosynthesis of the PPARα ligand from dietary fat. It is also possible that malonyl-CoA suppresses FAO in the liver of both types of mice. Obese patients with NAFLD manifest impaired suppression of DNL during fasting compared with obese individuals without NAFLD (8). Likewise, suppression of hepatic DNL during fasting might be impaired in *ob/ob* and Mc4r-KO mice, with the result that hepatic FASN deficiency promotes the accumulation of malonyl-CoA (more profoundly in *ob*/*ob* mice than in Mc4r-KO mice according to the extent of the enhancement of DNL), which allosterically inhibits mitochondrial CPT1α and thereby suppresses FAO (54). This malonyl-CoA–dependent mechanism could explain PPARα-independent suppression of FAO in Mc4r-KO HKO mice and may also contribute to suppression of FAO in ZFD-fed HKO mice. Given that dietary fructose markedly promotes hepatic DNL (55, 56), mice fed the ZFD, which contains a high proportion of sucrose (62% by weight), might experience some level of enhancement of hepatic DNL similar to genetically obese mice. The reversal of the metabolic phenotype of ZFD-fed HKO mice by switching to an NCD may thus be attributable not only to fat intake but also to DNL suppression that results from the interruption of sucrose intake and leads to release of the malonyl-CoA–dependent inhibition of FAO.

In Mc4r-KO mice, hepatic FASN deficiency ameliorated hyperglycemia in the fed state. In contrast, in *ob/ob* mice, loss of hepatic FASN resulted in marked exacerbation of fed hyperglycemia despite a greater suppression of gluconeogenesis compared with that apparent in Mc4r-KO mice. Hepatic glycogen accumulation and citrate-mediated inhibition of glycolysis appear to be key to the mechanism underlying this difference. In the liver of *ob/ob* F/F mice, after glycogen accumulation, dietary glucose is metabolized predominantly by glycolysis, the TCA cycle, and DNL (Figure 8C). In contrast, in the liver of *ob/ob* HKO mice, glycolysis might be suppressed as a result of citrate-mediated allosteric inhibition of PFK. The suppression of glycolysis together with glycogen accumulation may impair hepatic glucose utilization and uptake, leading to hyperglycemia as a result of glucose spillover (Figure 8, D and E). The fact that hepatic FASN deficiency exacerbates fed hyperglycemia only on the *ob/ob* genetic background may be explained by the increased hepatic citrate levels apparent in *ob*/*ob* HKO mice but not in Mc4r-KO HKO mice. Glucose flux into glycolysis, the TCA cycle, and DNL might be enhanced to a greater extent in *ob/ob* mice, with the result that hepatic FASN deficiency leads to citrate overproduction that exceeds the processing capacity of the TCA cycle. The loss of leptin signaling independent of the Mc4r pathway or the more pronounced hepatic insulin resistance in *ob/ob* mice may also affect the turnover of the TCA cycle.

Inhibition of hepatic DNL would be expected to terminate the vicious cycle linking this process and insulin resistance as well as to ameliorate both NAFLD and T2D. However, targeting of individual hepatic lipogenic enzymes other than FASN has failed to simultaneously alleviate these diseases. Hepatic ACLY depletion in *db/db* mice (57) or mice fed a high-sucrose, high-fat diet (HSHFD) (58) thus ameliorated NAFLD but had no effect on impaired glucose metabolism, whereas ablation of hepatocyte ACC1 in HSHFD-fed mice affected neither NAFLD nor glucose metabolism (59). Inhibition of ACC1 as well as mitochondrial ACC2, which produces mitochondrial malonyl-CoA and thereby inhibits FAO, was found to ameliorate NAFLD and improve glucose metabolism through suppression of DNL, enhancement of FAO, and promotion of insulin signaling in the liver (60, 61). However, this dual inhibition promoted hypertriglyceridemia (61, 62). We have now shown that hepatic FASN deficiency ameliorated NAFLD and diabetes in Mc4r-KO mice by suppressing DNL, FAO, and gluconeogenesis and improving hepatic insulin signaling without inducing hypertriglyceridemia. HFD-fed Mc4r-KO mice, but not HFD-fed *ob/ob* mice, progress from NAFL to NASH and hepatocellular carcinoma in association with the development of obesity and insulin resistance (51). Mc4r-KO mice may recapitulate the liver pathology of human obesity-related metabolic disorders. Inhibition of hepatic FASN may therefore be a potential therapeutic strategy for obesity-associated NAFLD and T2D in humans. Hepatic FAO is increased in individuals with NAFLD, which may give rise to oxidative stress and liver dysfunction (63, 64) that promote the progression to NASH and hepatocellular carcinoma. Hepatic FASN inhibition may also prevent such severe outcomes through suppression of FAO. Administration of the FASN inhibitor TVB-2640 for 10 days to obese men with metabolic syndrome suppressed hepatic DNL and reduced hepatic triglyceride content but had no effect on blood glucose, fat oxidation, or plasma triglyceride levels (65). Taking into account the etiology of obesity, it should be investigated whether chronic hepatic FASN inhibition improves glycemic control in T2D as well as NAFLD. On the other hand, as observed in *ob*/*ob* mice, targeting of hepatic FASN in obese individuals may exacerbate hyperglycemia and liver dysfunction, despite an associated amelioration of hepatic steatosis and improvement in glucose tolerance. Given that FASN expression is upregulated in cancer cells and contributes to their proliferation and survival (66), FASN inhibition is also a potential therapeutic strategy for cancer (67, 68). Administration of an FASN inhibitor to obese cancer patients with T2D, NAFLD, and pronounced leptin insufficiency, however, should take into account the possibility of an induced deterioration of glycemic control and liver function.

With regard to limitations of our study, we cannot exclude the possibility that pathways other than hepatic gluconeogenesis — such as glycogenolysis, glycolysis, and glucose cycling in the liver as well as glucose uptake in extrahepatic organs — may contribute to the improved glucose metabolism induced by hepatic FASN deficiency in the mouse obesity models. Further investigations that include glucose clamp and flux analyses in the liver are required to clarify the possible role of these pathways. Hepatic FASN deficiency may also alter lipid profiles and lipoprotein metabolism, and such effects may contribute to the fasting hypercholesterolemia apparent in *ob/ob* HKO mice and to the amelioration of hepatic steatosis in *ob/ob* HKO and Mc4r-KO HKO mice. Studies that involve lipidome and lipoprotein metabolism analyses should provide insight into these possibilities.

## Methods

Supplemental Methods are available online with this article.

### Statistics.

Quantitative data are presented as means + SEM. No statistical method was used to predetermine sample size, which was based on preliminary data and previous studies. Each experiment was performed at least 3 times. Mice were excluded from experiments if they showed any sign of morbidity. The statistical significance of differences in mean values was determined with the 2-tailed Student’s *t* test or Welch’s *t* test for comparisons between 2 groups and by 1-way ANOVA and Tukey’s or Bonferroni’s test for comparisons among 3 or more groups. A *P* value of less than 0.05 was considered statistically significant. Data analysis was performed with Prism software version 6 (GraphPad Software).

### Study approval.

All mouse experiments were approved by the Institutional Animal Care and Use Committee of the NCGM (Tokyo, Japan) and were performed according to the approved procedures.

### Data availability.

All data in the manuscript and supplemental material presented in graphs and tables are provided in Supporting Data Values. Any additional information required to reanalyze the data reported in this paper is available from the corresponding author upon request.

## Author contributions

M Matsumoto and MK conceptualized the study. Y Inaba, HI, KY, MU, TK, NK, HUK, Y Kaburagi, SA, Y Kido, HS, and YT designed the methodology. TM, TY, TU, MS, M Mitsushima, TN, HY, KY, YN, HS, and M Matsumoto performed experiments. TM and M Matsumoto validated the data. KN, HN, AA, Y Izumida, NY, MI, and YO provided resources. TM, TY, MS, TU, MK, and M Matsumoto wrote the original draft of the manuscript. All authors reviewed and edited the manuscript. SM, KT, KU, MK, and M Matsumoto supervised the study. MS, M Mitsushima, Y Kaburagi, MK, and M Matsumoto acquired funding for the study.

## Supplementary Material

Supplemental data

Supporting data values

## Version 1 09/08/2023

Electronic publication
