# Supplementary material for: Hepatic FASN deficiency differentially affects nonalcoholic fatty liver disease and diabetes in mouse obesity models
Source: JCI Insight. 2023 Sep 8;8(17):e161282. doi: 10.1172/jci.insight.161282 (PMC10544238; doi:10.1172/jci.insight.161282)

SUPPLEMENTAL MATERIAL

Hepatic FASN deficiency differentially affects nonalcoholic fatty liver disease and diabetes in mouse obesity models

Toshiya Matsukawa, Takashi Yagi, Tohru Uchida, Mashito Sakai, Masaru Mitsushima, Takao Naganuma, Hiroyuki Yano, Yuka Inaba, Hiroshi Inoue, Keisuke Yanagida, Masaaki Uematsu, Kazuki Nakao, Harumi Nakao, Atsu Aiba, Yoji Nagashima, Tetsuya Kubota, Naoto Kubota, Yoshihiko Izumida, Naoya Yahagi, Hiroyuki Unoki-Kubota, Yasushi Kaburagi, Shun-ichiro Asahara, Yoshiaki Kido, Hideo Shindou, Michiko Itoh, Yoshihiro Ogawa, Shiro Minami, Yasuo Terauchi, Kazuyuki Tobe, Kohjiro Ueki, Masato Kasuga, and Michihiro Matsumoto

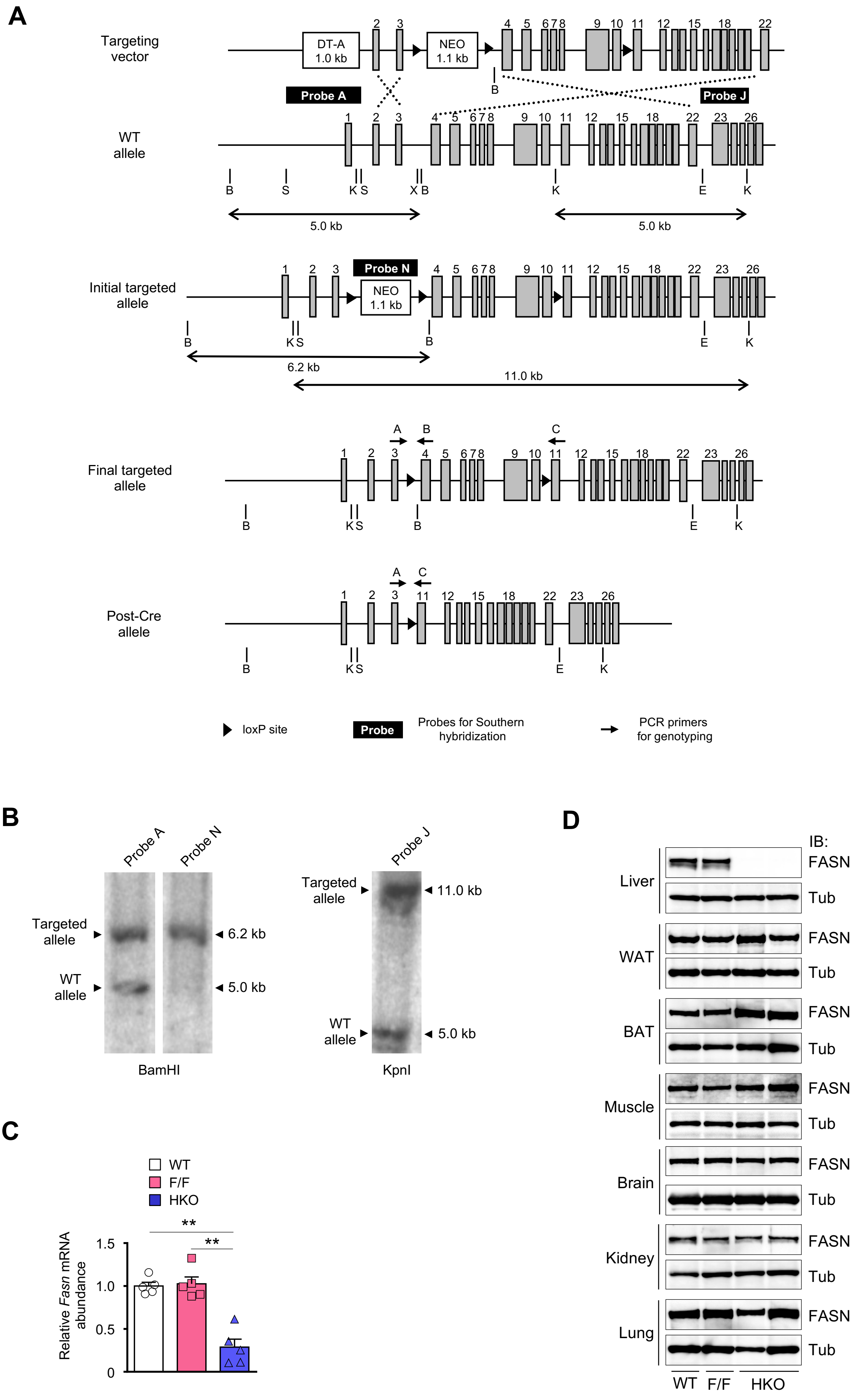
A
Targeting vector
DT-A 1.0 kb
NEO 1.1 kb
Probe A
Probe J
WT allele
5.0 kb
5.0 kb
Initial targeted allele
Probe N
6.2 kb
11.0 kb
Final targeted allele
Post-Cre allele
loxP site
Probe
Probes for Southern hybridization
PCR primers for genotyping
B
Probe A
Probe N
Targeted allele
WT allele
6.2 kb
5.0 kb
BamHI
Probe J
11.0 kb
5.0 kb
KpnI
C
WT
F/F
HKO
Relative Fasn mRNA abundance
D
IB:
Liver
WAT
BAT
Muscle
Brain
Kidney
Lung
FASN
Tub
WT
F/F
HKO

Supplemental Figure 1. Targeting of *Fasn* and generation of HKO mice. (**A**) Targeting vector, WT allele, and loxP-containing *Fasn* alleles before and after recombination. The post-Cre allele represents the *Fasn* allele after deletion of exons 4 to 10. DT-A and NEO denote a diphtheria toxin–A cassette for negative selection and a neomycin resistance cassette for positive selection, respectively. Gray boxes and closed arrowheads represent exons and loxP sites, respectively, and arrows A to C indicate genotyping primers (primer A, 5′-CAGCTAT-GAAGCAATTGTGGATGG-3′; primer B, 5′-AGTGGGACAGGGTTGTTGCCAAGC-3′; primer C, 5′-GAAGTCAGTAGGTCGATGAGGGC-3′). Probes A, N, and J are for Southern hybridization. B, BamHI site; S, SmaI site; K, KpnI site; X, XhoI site; E, EcoRV site. (**B**) Southern blot analysis of DNA from embryonic stem cells harboring a *Fasn* allele targeted by the targeting vector (initial targeted allele) and a WT allele. Genomic DNA was digested with BamHI and hybridized with external probe A (DNA fragment between two SmaI sites in the 5′ region of *Fasn*) or probe N (NEO cassette), or was digested with KpnI and hybridized with external probe J (DNA fragment between EcoRV and KpnI sites in the 3′ region of *Fasn*). (**C**) RT-qPCR analysis of *Fasn* mRNA in the liver of 10-wk-old WT, F/F (control), and HKO mice. Data are means + SEM (n = 5 mice). $^{**}P < 0.01$ (one-way ANOVA followed by Tukey's multiple comparison test). (**D**) Immunoblot (IB) analysis of FASN and α-tubulin (Tub, loading control) in various tissues of 10-wk-old WT, F/F, and HKO mice. WAT, white adipose tissue; BAT, brown adipose tissue.

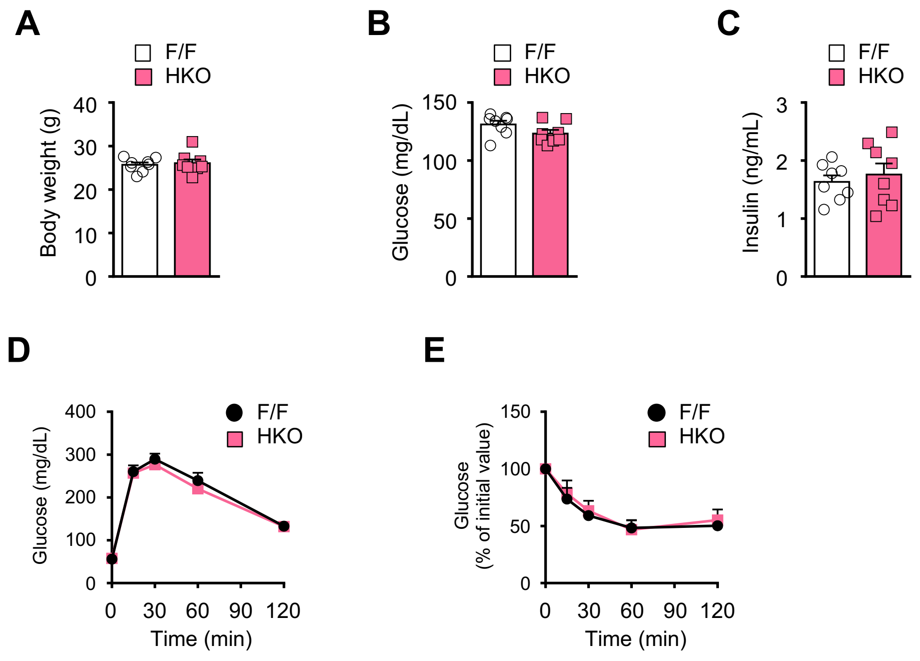

Supplemental Figure 2. Metabolic characteristics of 12-wk-old NCD-fed HKO mice. (**A**–**C**) BW (**A**) as well as blood glucose (**B**) and plasma insulin (**C**) concentrations in the fed state for HKO mice and F/F littermate controls (n = 8). (**D** and **E**) An IPGTT (2 g of glucose per kilogram of BW) (**D**) and ITT (0.75 U of human regular insulin per kilogram of BW) (**E**) for HKO mice and littermate controls (n = 4 or 5). All data are means + SEM for the indicated numbers (n) of mice.

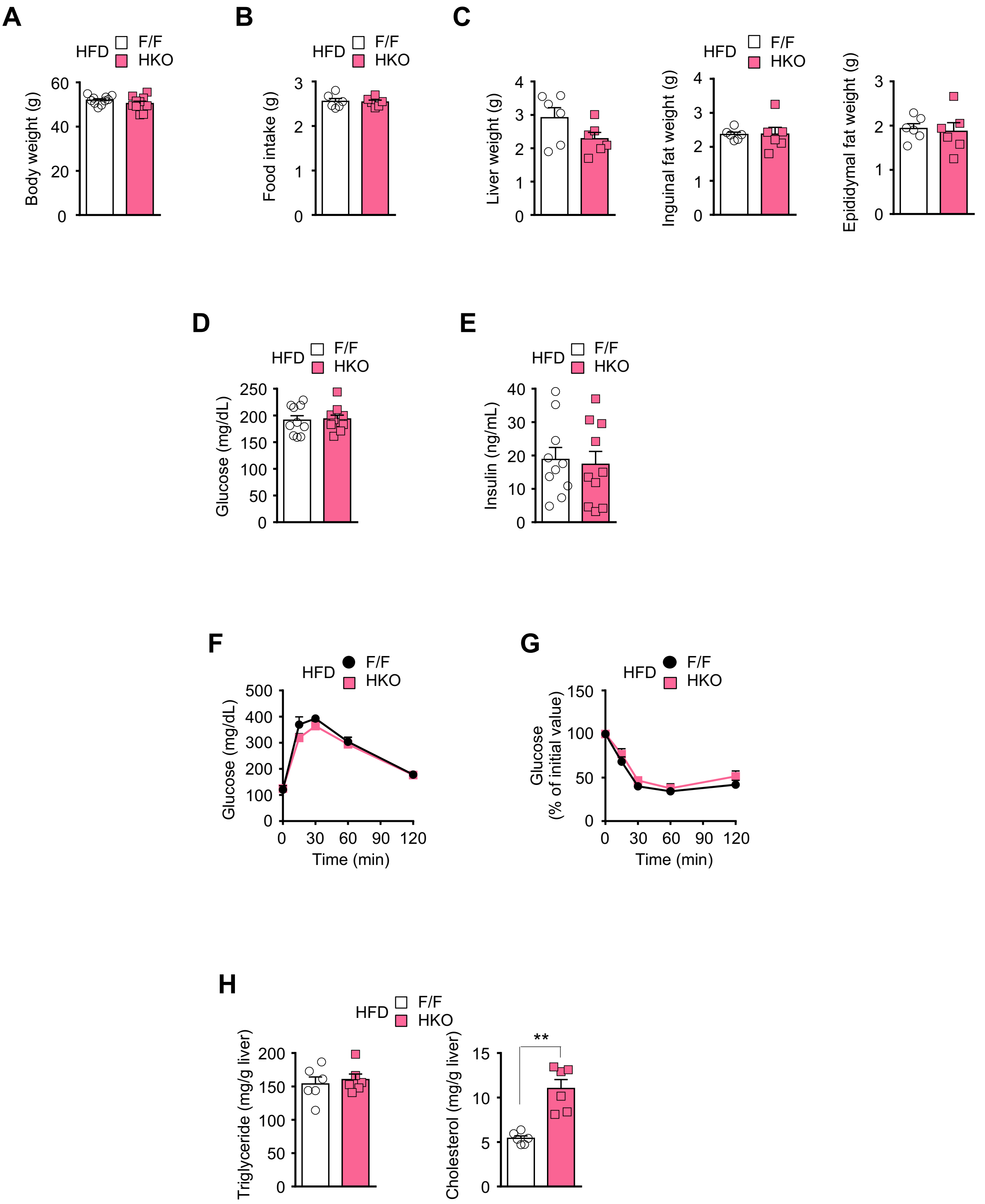

Supplemental Figure 3. Metabolic characteristics of 24-wk-old HFD-fed HKO mice. (**A**–**C**) BW (*n* = 10) (**A**), food intake over 24 h (*n* = 6) (**B**), and weight of the liver, inguinal fat, and epididymal fat (*n* = 6) (**C**) for F/F and HKO mice maintained on an HFD. (**D** and **E**) Blood glucose (**D**) and plasma insulin (**E**) concentrations in the fed state for HKO mice and littermate controls (*n* = 10). (**F** and **G**) An IPGTT (1.5 g of glucose per kilogram of BW) (**F**)

and ITT (2 U of human regular insulin per kilogram of BW) (**G**) for HKO mice and littermate controls (*n* = 5 or 6). (**H**) Hepatic triglyceride and cholesterol levels in the fed state for HKO mice and littermate controls (*n* = 6). All data are means + SEM for the indicated numbers of mice. ***P* < 0.01 (two-tailed Student's *t* test).

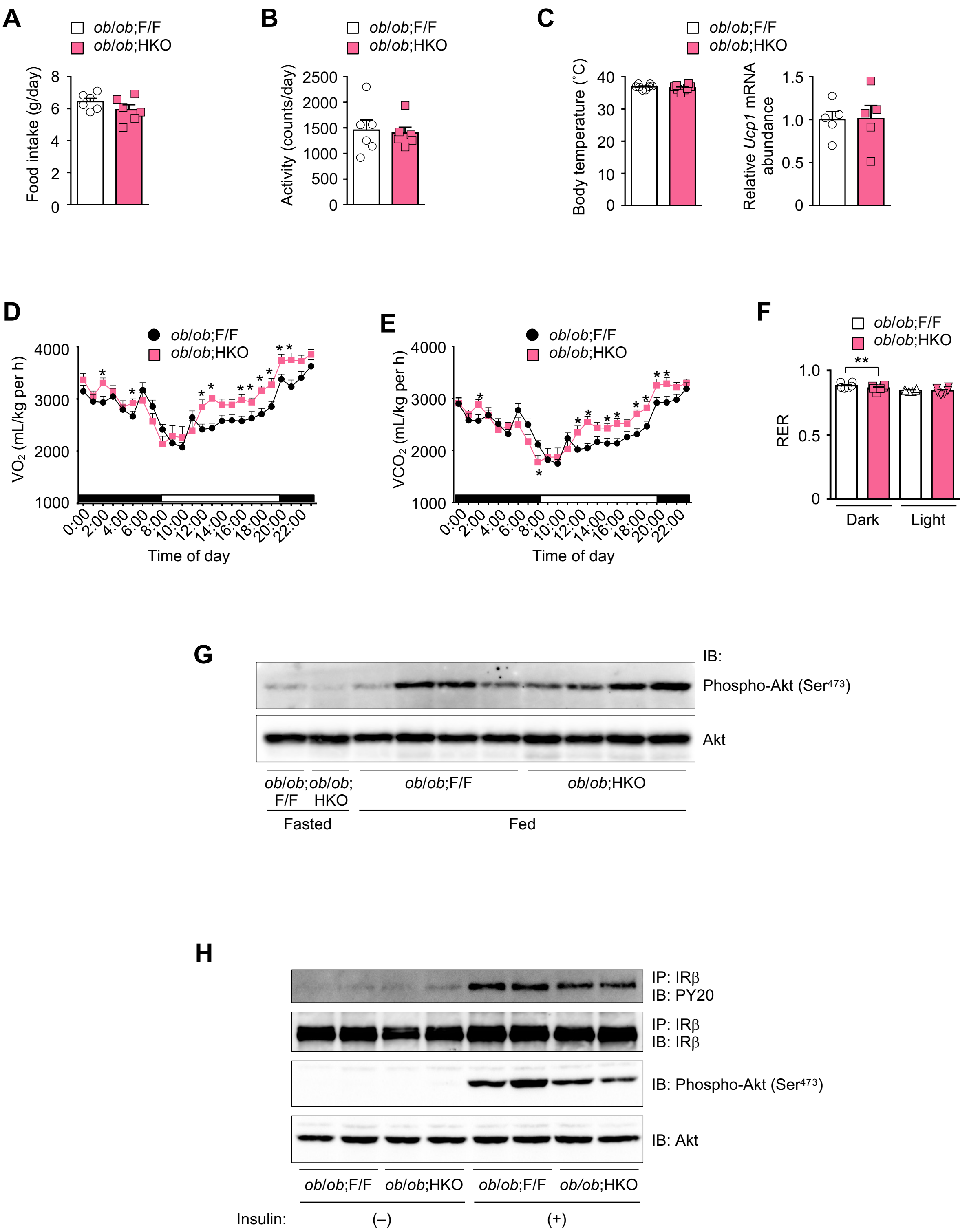

Supplemental Figure 4. Effects of hepatic FASN deficiency on energy metabolism and hepatic insulin signaling in NCD-fed *ob/ob* mice. (**A** and **B**) Food intake (**A**) and locomotor activity (**B**) over 24 h for *ob/ob*;F/F and *ob/ob*;HKO mice at 10 wk of age (*n* = 6). (**C**) Body temperature (*n* = 8) and RT-qPCR analysis of *Ucp1* mRNA in brown adipose tissue (*n* = 5) for mice at 10 wk of age. (**D**–**F**) Oxygen consumption (VO_2) (**D**) and carbon dioxide

production (VCO_2) (**E**) over 24 h as well as the respiratory exchange ratio (RER) in the dark and light phases (**F**) for mice at 8- to 12-wk of age (*n* = 5 or 6). (**G**) Immunoblot analysis of Ser^{473}-phosphorylated and total forms of Akt in the liver of 10-wk-old mice in the fasted and fed states. (**H**) Effects of insulin on IRβ and Akt phosphorylation in the liver of 8-wk-old mice. Mice deprived of food overnight were injected intravenously with insulin (5 U/kg) or vehicle (–), 2 min after which the liver was isolated, lysed, and subjected to immunoprecipitation (IP) with antibodies to IRβ. The resulting precipitates were subjected to immunoblot analysis with antibodies to phosphotyrosine (PY20) or to IRβ. Alternatively, the liver lysates were subjected directly to immunoblot analysis with antibodies to Ser^{473}-phosphorylated or total forms of Akt. Each lane in **G** and **H** corresponds to one mouse. All quantitative data are means + SEM for the indicated numbers of mice. $^{*}P < 0.05$, $^{**}P < 0.01$ compared with *ob/ob*;F/F mice or as indicated (two-tailed Student's *t* test).

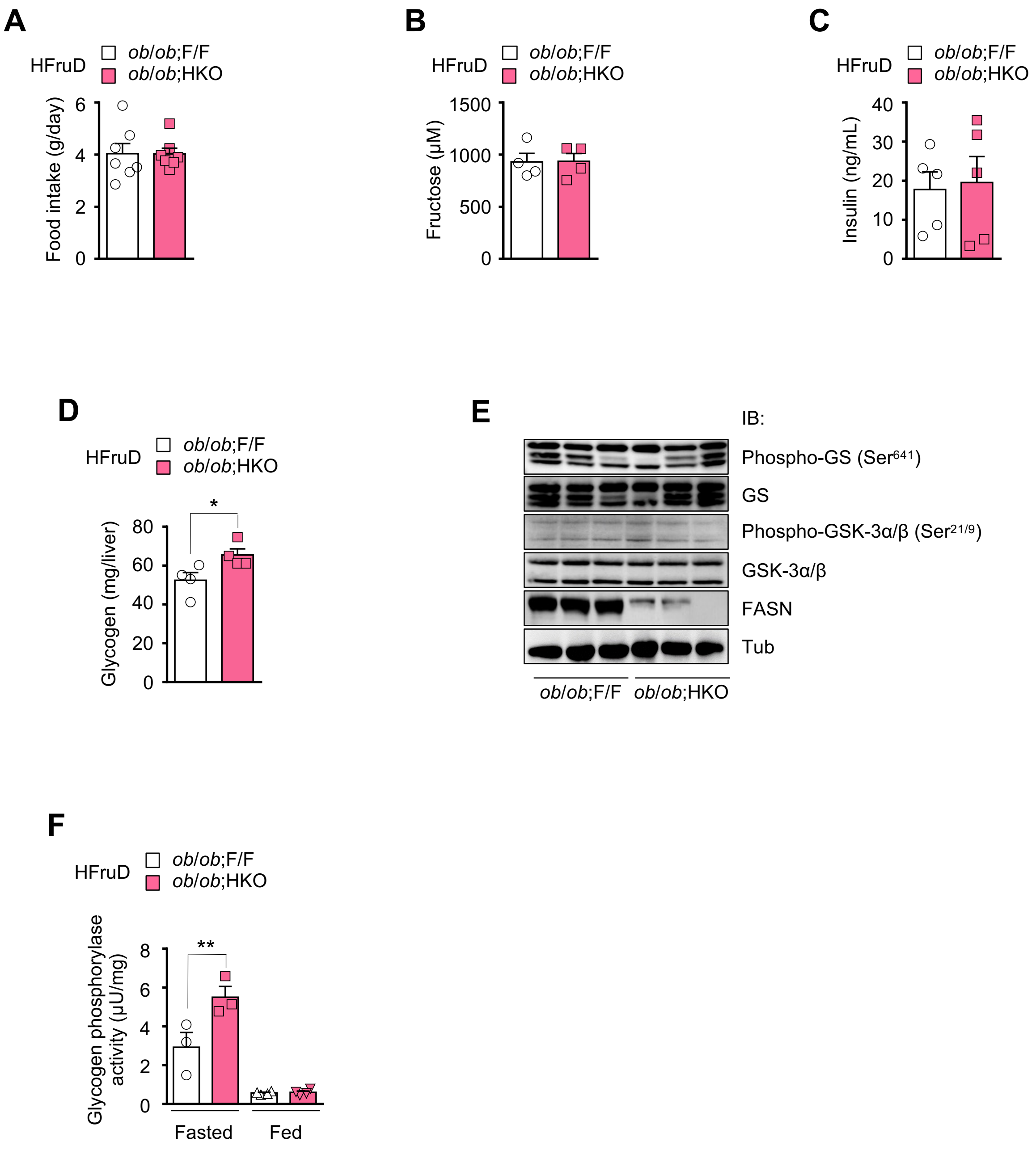

Supplemental Figure 5. Effects of an HFruD on the metabolic phenotype of 24-wk-old *ob/ob*;HKO mice. (**A**) Food intake over 24 h for *ob/ob*;F/F and *ob/ob*;HKO mice maintained on an HFruD ($n = 7$). (**B** and **C**) Plasma fructose ($n = 4$) (**B**) and insulin ($n = 5$) (**C**) concentrations in the fed state for HFruD-fed mice. (**D**) Hepatic glycogen content (mg/liver) in the fed state for HFruD-fed mice ($n = 4$). (**E**) Immunoblot analysis of phosphorylated and total forms of GS and GSK-3α/β in the liver of HFruD-fed mice in the fed state ($n = 3$). (**F**) Hepatic glycogen phosphorylase activity in the fed or fasted state for HFruD-fed mice ($n = 3$ or 4). All quantitative data are means + SEM for the indicated numbers of mice. $*P < 0.05$,

$P < 0.01$ [two-tailed Student's *t* test (D**) or one-way ANOVA followed by Tukey's multiple comparison test (**F**)].

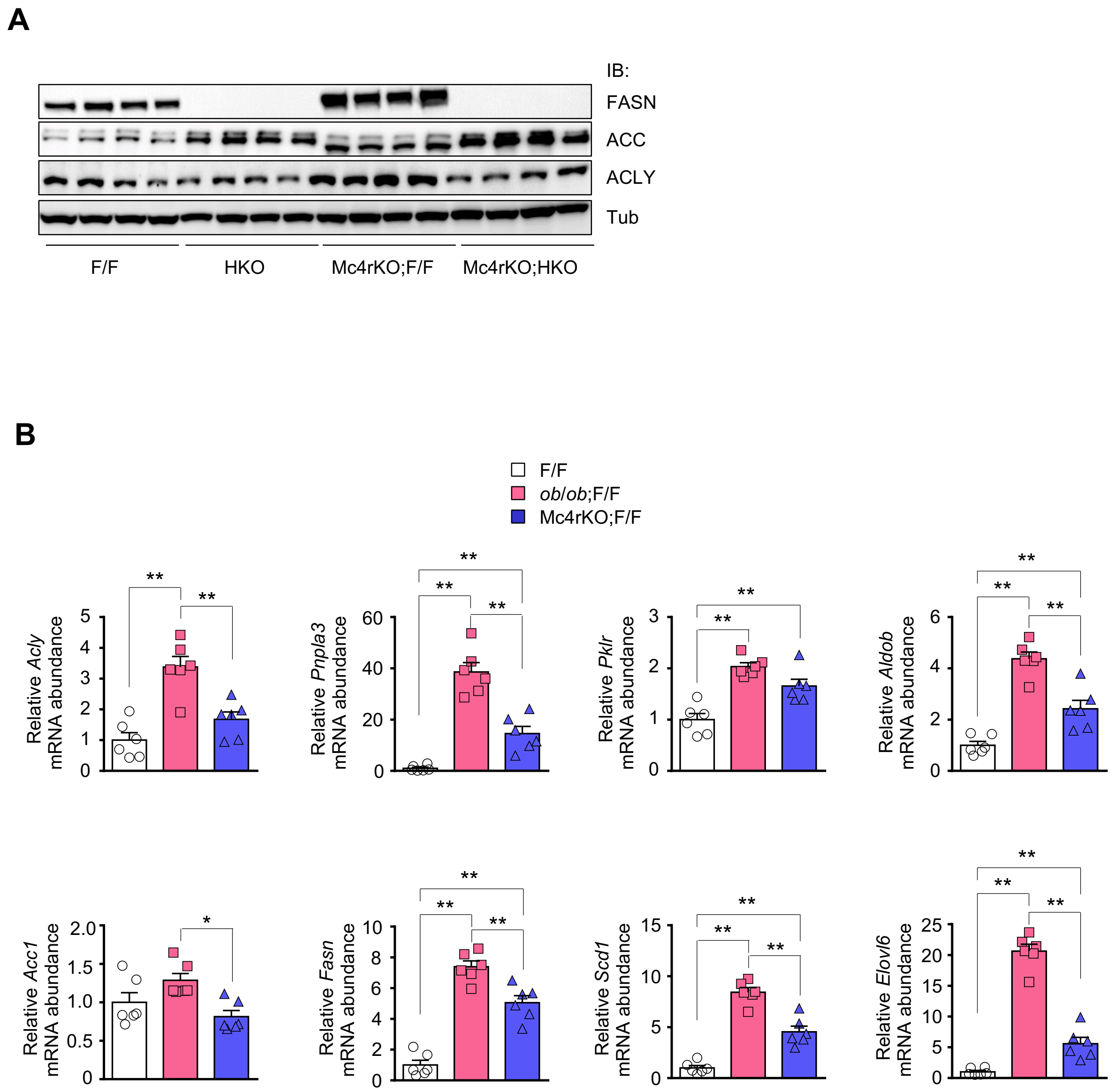

Supplemental Figure 6. Hepatic expression of lipogenic enzymes in F/F, HKO, Mc4rKO;F/F, and Mc4rKO;HKO mice as well as of SREBP1c and ChREBP target genes in F/F, *ob/ob*;F/F, and Mc4rKO;F/F mice. (**A**) Immunoblot analysis of FASN, ACC, and ACLY in the liver of NCD-fed 10-wk-old F/F and HKO mice and 16- to 20-wk-old Mc4rKO;F/F and Mc4rKO;HKO mice in the fed state. Each lane corresponds to one mouse. (**B**) RT-qPCR analysis of target genes of SREBP1c, ChREBP, or both transcription factors in the liver of 10-wk-old F/F and *ob/ob*;F/F mice as well as of 22- to 24-wk-old Mc4rKO;F/F mice in the fed state. *Elovl6*, elongation of long-chain fatty acids family member 6 gene; *Pnpla3*, patatin-like phospholipase domain–containing protein 3 gene; *Scd1*, stearoyl-CoA desaturase 1 gene. Data are means + SEM for six mice. *$P < 0.05$, **$P < 0.01$ (one-way ANOVA followed by Tukey's multiple comparison test).

Hepatic metabolite abundance (nmol/g) in *ob/ob*;F/F and *ob/ob*;HKO mice in the fasted state

Metabolite	*ob/ob*;F/F	*ob/ob*;HKO	*P* value
2-Phosphoglycerate	0.85±0.08	0.23±0.11	0.0037[a]
3-Phosphoglycerate	7.70±0.92	3.90±0.41	0.0086[a]
Acetyl-CoA	6.18±1.02	3.13±0.87	0.0481[b]
Alanine	1789.25±153.57	2399.00±155.53	0.0261[a]
Aconitate	0.34±0.17	0.26±0.19	0.3955[a]
Citrate	66.50±15.43	70.75±10.29	0.4246[a]
Dihydroxyacetone phosphate	63.75±10.18	4.20±2.00	0.0065[a]
Fructose 1,6-bisphosphate	7.98±2.05	2.43±0.53	0.0320[b]
Fructose 6-phosphate	65.75±9.01	30.50±5.44	0.0136[b]
Fumarate	56.75±8.14	59.50±10.63	0.4324[a]
Glucose 1-phosphate	16.00±2.37	7.65±1.17	0.0169[b]
Glucose 6-phosphate	228.75±28.95	99.50±15.45	0.0072[b]
Glycerol 3-phosphate	1782.75±135.65	2131.25±135.38	0.0586[b]
Lactate	11627.25±715.63	8734.50±338.35	0.0097[a]
Malate	30.25±19.88	3.50±3.03	0.1647[a]
Phosphoenolpyruvate	0.46±0.20	0.00±0.00	0.0229[a]
Pyruvate	207.75±13.08	219.75±22.15	0.3501[a]
Succinate	489.75±78.16	437.75±65.07	0.3367[a]

Data are means ± SEM (*n* = 4 mice).
[a], Analyzed by Welch's *t* test.
[b], Analyzed by Student's *t* test.

Supplemental Table 1

Hepatic metabolite abundance in *ob/ob*;F/F and *ob/ob*;HKO mice in the fed state

Metabolite	*ob/ob*;F/F	*ob/ob*;HKO	*P* value
2-Phosphoglycerate	0.93±0.06	0.48±0.16	0.0343[a]
3-Phosphoglycerate	7.95±0.40	4.53±0.69	0.0050[a]
6-Phosphogluconate	19.75±0.96	34.00±3.82	0.0222[b]
Acetyl-CoA	20.13±5.63	36.33±2.84	0.0519[a]
Citrate	53.17±6.05	121.00±24.42	0.0259[b]
Dihydroxyacetone phosphate	155.25±22.96	73.25±8.41	0.0136[a]
Fructose 1,6-bisphosphate	168.67±32.35	82.00±11.09	0.0537[a]
Fructose 6-phosphate	101.00±7.44	202.75±22.00	0.0045[a]
Fumarate	740.00±16.66	1436.00±74.55	0.0009[a]
Glucose 1-phosphate	27.75±2.36	50.00±5.23	0.0076[a]
Glucose 6-phosphate	380.50±25.65	610.00±40.31	0.0030[a]
Glyceraldehyde 3-phosphate	4.20±0.65	3.33±0.21	0.1561[a]
Isocitrate	8.47±0.59	16.33±2.33	0.0277[a]
Malate	325.00±9.42	500.00±28.02	0.0042[a]
Malonyl-CoA	N/A	13.67±8.46	0.1590[b]
N-acetylglucosamine	$4.60 \times 10^{-5} \pm 3.70 \times 10^{-6}$ *	$6.80 \times 10^{-5} \pm 4.10 \times 10^{-6}$ *	0.0030[b]
N-acetylglucosamine 1-phosphate	$8.30 \times 10^{-5} \pm 1.60 \times 10^{-5}$ *	$1.90 \times 10^{-4} \pm 4.80 \times 10^{-5}$ *	0.0500[b]
N-acetylglucosamine 6-phosphate	$6.40 \times 10^{-5} \pm 1.50 \times 10^{-5}$ *	$1.20 \times 10^{-4} \pm 4.50 \times 10^{-6}$ *	0.0200[b]
Phosphoenolpyruvate	0.27±0.12	0.00±0.00	0.0509[a]
Pyruvate	230.25±6.79	328.75±66.88	0.1462[b]
Ribose 5-phosphate	13.25±1.95	21.50±2.36	0.0291[a]
Ribulose 5-phosphate	4.58±0.73	13.00±0.50	0.0001[a]
Succinate	395.25±100.77	750.50±54.03	0.0180[a]
UDP-glucose	135.75±6.78	198.75±4.99	0.0003[a]
UDP-*N*-acetylglucosamine	$3.50 \times 10^{-3} \pm 7.10 \times 10^{-5}$ *	$6.00 \times 10^{-3} \pm 1.40 \times 10^{-4}$ *	0.0001[b]

Data are means ± SEM (*n* = 3-6 mice)and are expressed as nmol/g or *relative area/mg.
N/A, not available,
[a], Analyzed by Student's *t* test.
[b], Analyzed by Welch's *t* test.

Supplemental Table 2

Diet composition

	High-fat diet (HFD)	High-fructose diet (HFruD)
**Composition (g%)**		
Protein	26	20
Fat	35	5
Carbohydrate	26	66
**Energy (kcal/g)**	3.90	4.82
**Ingredient (g%)**		
Casein	25.87	20.00
DL-Methionine	—	0.30
L-Cystine	0.35	—
Soybean oil	3.23	5.00
Lard	31.69	—
Bovine fat	—	—
Corn starch	0.00	5.00
Sucrose	8.90	—
Fructose	—	60.00
Maltodextrin	16.17	—
Cellulose	5.83	5.00
**Vendor (Product #)**	Research Diets (D12492)	Research Diets (D00111301)

	Normal chow diet (NCD)
**Composition (g%)**	
Crude protein	25.10
Crude fat	4.51
Crude fiber	5.05
Crude ash	6.97
Nitrogen free extract	49.72
**Energy (kcal/g)**	3.40
**Vendor (Product #)**	CLEA Japan (CE-2)

Supplemental Table 3

Antibodies used for immunoprecipitation (IP) and immunoblot (IB) analysis

Antigen	Dilution	Cat#	Vendor
Acetyl-CoA carboxylase	1:1000 IB	#3662	Cell Signaling, Danvers, MA
Phospho-acetyl-CoA carboxylase (Ser^{79})	1:1000 IB	#3661	Cell Signaling, Danvers, MA
Akt (pan)	1:1000 IB	#4691	Cell Signaling, Danvers, MA
Phospho-Akt (Ser^{473})	1:1000 IB	#9271	Cell Signaling, Danvers, MA
AMPKα	1:1000 IB	#2532	Cell Signaling, Danvers, MA
Phospho-AMPKα (Thr^{172})	1:1000 IB	#2531	Cell Signaling, Danvers, MA
ATP citrate lyase	1:1000 IB	#4332	Cell Signaling, Danvers, MA
Fatty acid synthase (H-300)	1:500 IB	sc-20140	Santa Cruz, Dallas, TX
Glycogen synthase	1:1000 IB	#3886	Cell Signaling, Danvers, MA
Phospho-glycogen synthase (Ser^{641})	1:1000 IB	#47043	Cell Signaling, Danvers, MA
GSK-3α/β	1:1000 IB	#5676	Cell Signaling, Danvers, MA
Phospho-GSK-3α/β ($Ser^{21/9}$)	1:1000 IB	#9331	Cell Signaling, Danvers, MA
Insulin receptor β	1:100 IP; 1:500 IB	sc-711	Santa Cruz, Dallas, TX
Phospho-insulin receptor β (Tyr^{1150})	1:1000 IB	#3918	Cell Signaling, Danvers, MA
Phosphotyrosine (PY20)	1:1000 IB	sc-508	Santa Cruz, Dallas, TX
Raptor (24C12)	1:1000 IB	#2280	Cell Signaling, Danvers, MA
Phospho-Raptor (Ser^{792})	1:1000 IB	#2083	Cell Signaling, Danvers, MA
α-Tubulin	1:5000 IB	T6199	Sigma, St. Louis, MO

Supplemental Table 4

Primers for RT-qPCR analysis

Gene	Forward	Reverse
Acc1	5'-GGACAGACTGATCGCAGAGAAAG-3'	5'-GCTGTTCCTCAGGCTCACAT-3'
Acly	5'-GCCAGCGGGAGCACATC-3'	5'-CTTTGCAGGTGCCACTTCATC-3'
Acox1	5'-GTGCAGCTCAGAGTCTGTCCAA-3'	5'-TACTGCTGCGTCTGAAAATCCA-3'
Aldob	5'-TGTCCTTCCAGCCTTGCTATCC-3'	5'-AGTGCTCCAGGTCATGGTCTCC-3'
Atf4	5'-GTCTCCTCGGCCCAAACCTTAT-3'	5'-TGGCTGCTGTCTTGTTTTGCTC-3'
Bax	5'-TGAAGACAGGGGCCTTTTTG-3'	5'-AATTCGCCGGAGACACTCG-3'
Ccl2	5'-GCAGGTGTCCCAAAGAAGCTGT-3'	5'-TCATTTGGTTCCGATCCAGGTT-3'
Cpt1a	5'-CGCACGGAAGGAAAATGGAC-3'	5'-GGAGCCAAGGCATCTCTGGA-3'
Ddit3	5'-CCTCGCTCTCCAGATTCCAGTC-3'	5'-TGGGCACTGACCACTCTGTTTC-3'
Elvol6	5'-CCCATTTCCTGGTGCCTGAG-3'	5'-TGGCGGCCCAATGACTAACT-3'
Fasn	5'-GAGTTCTCAGGCCGGGATAGGT-3'	5'-TGGTATAGACGACGGGCACAGA-3'
Gck	5'-GACTGTGGACCAGGGTGTTGTG-3'	5'-TCGTCAGAGTGGCAATTTTGGA-3'
Gys2	5'-GGACTGGAGATACCTGGGCAGA-3'	5'-CTGAGGAGGGCCTGGGATACTT-3'
G6pc	5'-CTGTCCCGGATCTACCTTGCTG-3'	5'-AATACTTCCGGAGGCTGGCATT-3'
Hmgcs2	5'-TGCAGGAAACTTCGCTCACA-3'	5'-AAATAGACCTCCAGGGCAAGGA-3'
Il6	5'-TAGTCCTTCCTACCCCAATTTCC-3'	5'-ATGGTCTTGGTCCTTAGCC-3'
Pck1	5'-CCTGGAAGAACAAGGAGTGG-3'	5'-AGGGTCAATAATGGGGCACT-3'
Pfkl	5'-GGAGGTGACTCTGGCCTCTCAG-3'	5'-GTATCGAAGCCCAGCCTCTGAA-3'
Pklr	5'-AGGCTCTCGGCTGAACATCATC-3'	5'-CCACCCACACTGTCTTTGCATC-3'
Pmaip1	5'-GCAGAGCTACCACCTGAGTTC-3'	5'-CTTTTGCGACTTCCCAGGCA-3'
Pnpla3	5'-ATGGCAAACTTGTGGGAGAC-3'	5'-AGCCCTCCCTGTCCTTAATC-3'
Ppara	5'-CAAAACACCAGTGTGAATTAC-3'	5'-TAGGCAGTGCATCAGCGAAGG-3'
Ppargc1a	5'-CACAAAGACGTCCCTGCTCAGA-3'	5'-CCTTGGGGTCATTTGGTGACTC-3'
Ppp1r15a	5'-GGGTGGTCCAGCTGAGAATG-3'	5'-CAGGGGTGCTGGGTTTGTAT-3'
Scd1	5'-CCTCCTGCAAGCTCTACACC-3'	5'-CAGCCGAGCCTTGTAAGTTC-3'
Tnf	5'-CTATGGCCCAGACCCTCACACT-3'	5'-CACTCCAGCTGCTCCTCCACTT-3'
Ucp1	5'-CCTGGCAAAAACAGAAGGATTG-3'	5'-GGCTGAGATCTTGTTTCCGAGA-3'
Rn18s	5'-AATTCCCAGTAAGTGCGGGTCA-3'	5'-GATCCGAGGGCCTCACTAAACC-3'

Supplemental Table 5

Supplemental Methods

Generation of HKO mice. The targeting vector was designed to delete exons 4 to 10 of *Fasn* (Supplemental Figure 1A). A loxP sequence was inserted into the KpnI site of intron 10, a pMC1-neo cassette (1) flanked by loxP sequences was inserted into the BamHI site of intron 3, and a pMC1-DTA cassette (1) was inserted into the SmaI site of intron 1. Embryonic stem cells appropriately targeted by the vector and with loxP sequences flanking exons 4 to 10 of *Fasn* were used to generate chimeric mice, which were then mated with C57BL/6J mice to yield animals heterozygous for the targeted allele (*FloxP/+* mice). *FloxP/+* mice were crossed with mice expressing Cre recombinase under the control of the adenovirus *EIIa* promoter (*EIIa-cre*$^{+}$ mice). Male *FloxP/+;EIIa-cre*$^{+}$ mice, which showed various extents of mosaicism for the partially Cre-recombined floxed allele (2), were crossed with female C57BL/6J mice to yield mice lacking the *EIIa-cre*$^{+}$ allele and heterozygous for the targeted allele without the neo cassette, but with exons 4 to 10 flanked by loxP sequences (F/+ mice). These mice were crossed with mice harboring the Cre gene under the control of the mouse albumin gene promoter (*Alb-cre*$^{+}$ mice) (3) to generate mice heterozygous for the targeted allele and the *Alb-cre* allele (F/+;*Alb-cre*$^{+}$ mice). F/+;*Alb-cre*$^{+}$ mice were backcrossed onto the C57BL/6J genetic background for more than seven generations. Mice with hepatocyte-specific deletion of *Fasn* (F/F;*Alb-cre*$^{+}$, or HKO) and mice homozygous for the targeted allele with the flanking loxP sequences (F/F) as controls were then generated by mating F/+;*Alb-cre*$^{+}$ mice with F/+ mice. All possible genotypes were born at the expected frequency for Mendelian inheritance.

Mice and diets. Mice were maintained on a 12-h-light, 12-h-dark cycle with food and water provided ad libitum unless otherwise specified. B6.Cg-*Lep*^{ob}/J mice (*Lep*$^{ob/+}$ mice on the C57BL/6J background) were obtained from Charles River (Yokohama, Japan) and were crossed with HKO mice on the C57BL/6J background to obtain *Lep*$^{ob/+}$;F/+;*Alb-cre*$^{+}$ mice, which were then intercrossed to obtain *Lep*$^{ob/ob}$;F/F mice and *Lep*$^{ob/ob}$;HKO mice (*ob/ob*;F/F and *ob/ob*;HKO mice, respectively). Progeny from both strains were detected at the expected Mendelian frequency. We maintained F/F and HKO mice as well as *ob*/+;F/F and *ob/*+;HKO mice on the C57BL/6J background and used male mice for the present study. Mice heterozygous for a targeted allele of *Mc4r* on the C57BL/6J background (*Mc4r*$^{+/-}$ mice) were kindly provided by J. K. Elmquist (University of Texas Southwest Medical Center, Dallas, TX) (4) and were crossed with HKO mice on the C57BL/6J background to obtain *Mc4r*$^{+/-}$;F/+;*Alb-cre*$^{+}$ mice, which were then intercrossed to obtain *Mc4r*$^{-/-}$;F/F mice and *Mc4r*$^{-/-}$;HKO mice (Mc4rKO;F/F and Mc4rKO;HKO mice, respectively). Progeny from both

of these strains were also observed at the expected Mendelian frequency. We maintained Mc4rKO;F/F and Mc4rKO;HKO mice on the C57BL/6J background and used male mice for the present study. Mice were provided ad libitum access to either an NCD (CLEA Japan, CE-2), HFD (Research Diets, D12492), or HFruD (Research Diets, D00111301). The composition of the diets is shown in Supplemental Table 3. The animals were provided with the HFD or HFruD from 4 wk of age for 20 wk, or as indicated.

Chemicals and antibodies. Human regular insulin (Humulin R) and leptin were obtained from Eli Lilly (428021014) and PeproTech (20347-44), respectively. 2-DG and sodium pyruvate were from Sigma-Aldrich (D8375 and P8574, respectively). D(+)-Glucose and glycerol were from Nacalai Tesque (16805-35 and 17017-93, respectively). Details of the antibodies used are provided in Supplemental Table 4.

Biochemical assays. Blood glucose and plasma FFA concentrations were determined as described (5, 6). Plasma concentrations of insulin, fructose, and β-hydroxybutyrate were measured with a Mouse Insulin ELISA Kit (TMB) (Shibayagi, AKRIN-011T), EnzyChrom Fructose Assay Kit (BioAssay Systems, EFRU-100), and β-Hydroxybutyrate Colorimetric Assay Kit (BioVision, K632-100), respectively. The plasma levels of total cholesterol, triglyceride, and transaminases were determined with Cholesterol E-test Wako, Triglyceride E-test Wako, and Transaminase CII-test Wako kits, respectively (Fujifilm Wako Pure Chemical: 439-17501, 432-40201, and 431-30901, respectively).

Metabolic tests. For the IPGTT, mice deprived of food for 16 h were injected intraperitoneally with glucose (1.5 or 2 g/kg of BW as indicated). For the ITT, mice in the randomly fed state were injected intraperitoneally with human regular insulin (0.75, 2, or 4 U/kg of BW as indicated). For the pyruvate and glycerol tolerance tests, mice deprived of food for 16 h were injected intraperitoneally with pyruvate (2 g/kg of BW) or glycerol (3 g/kg of BW), respectively, dissolved in physiological saline. Blood glucose concentrations were measured at the indicated time points.

Metabolic measurements. Food intake and locomotor activity were determined with the use of a food intake, drinking, and locomotor activity monitoring system for mice (Shin Factory, MFD-100M and ACTIMO-100S). Oxygen consumption, carbon dioxide production, and the respiratory exchange ratio were measured every 5 min over 24 h under the resting condition with the use of an Oxymax V4.00 calorimeter (Columbus Instruments). Mice were monitored individually in these systems for 2 days (1 day of acclimation followed by 1 day of measurement). Body temperature was measured with a rectal probe attached to a digital thermometer (Physitemp Instruments, BAT-12).

Leptin supplementation. An osmotic pump (Alzet, Micro-osmotic Pump model 1002) was implanted subcutaneously into individually caged male *ob/ob*;F/F or *ob/ob*;HKO mice at 8 to 9 wk of age. PBS vehicle or leptin at 2.0 mg/day was administered for 7 days via the pump.

Histology. Mouse liver was excised, fixed in a 10% neutral formalin solution, embedded in paraffin, sectioned at a thickness of 4 to 5 mm, and subjected to H&E or PAS staining. Frozen sections of liver tissue prepared at a thickness of 20 μm in Neg-50 Frozen Section Medium (Thermo Fisher Scientific, 6402G) were subjected to oil red O staining. Paraffin embedding, sectioning, and H&E, PAS, and oil red O staining were performed at the Communal Laboratory, Research Institute, National Center for Global Health and Medicine.

Immunoprecipitation and immunoblot analysis. Tissue samples (~100 mg wet weight) were lysed in a solution containing 20 mM Tris-HCl (pH 7.5), 1% NP-40, 10% glycerol, 137 mM NaCl, 1 mM $MgCl_2$, 2.5 mM $CaCl_2$, 1 mM DTT, 1 mM Na_3VO_4, 1 mM EDTA, and protease and phosphatase inhibitors (Roche: 5056489001 and 4906837001, respectively). The lysates were subjected to immunoprecipitation with the indicated antibodies and Dynabeads Protein G (Veritas, DB10004), and the resulting precipitates were fractionated by SDS-PAGE. The separated proteins were transferred to a nitrocellulose membrane, which was then probed with primary antibodies. Immune complexes were visualized with Amersham ECL HRP-conjugated secondary antibodies (Cytiva, NA931 and NA934), SuperSignal West Pico Chemiluminescent Substrate (Thermo Fisher Scientific, 34580), and a Luminoimage Analyzer LAS-4000mini (Fujifilm).

Transduction of recombinant adenovirus vectors. A control adenovirus encoding β-galactosidase (LacZ) and an adenovirus encoding GS were described previously (7). Adenoviruses were injected into mice via the tail vein at a dose of 3.0×10^9 PFU, and the mice were analyzed in the fed or fasted state at 4 days after adenoviral transduction.

RT-qPCR analysis. Total RNA was extracted from pulverized liver with the use of an RNeasy Mini Kit and RNase-Free DNase Set (Qiagen: 74104 and 79254, respectively) or a NucleoSpin RNA Kit (Macherey Nagel, 740955). The RNA was subjected to RT with random primers and the use of a High Capacity cDNA Reverse Transcription Kit (Applied Biosystems, 4368813). The resulting cDNA was subjected to qPCR analysis in triplicate with the use of a StepOnePlus Real-Time PCR System and Power UP SYBR Green Master Mix (Applied Biosystems, A25742). Relative mRNA abundance was calculated by the standard curve method and with normalization according to the corresponding amount of 18S rRNA.

Primer sequences for qPCR are listed in Supplemental Table 5 and were described previously (8).

Metabolite measurement. Frozen liver tissue (~50 mg) was transferred to 1.5 mL of 50% acetonitrile solution in deionized water containing internal standards (Solution ID 304-1002; Human Metabolome Technologies, Yamagata, Japan) at 0ºC in order to inactivate enzymes. The tissue was homogenized three times at 1500 rpm for 120 s with a Micro Smash MS100R device (Tomy Digital Biology), and the homogenate was centrifuged at 2300 × *g* and 4ºC for 5 min. A portion (800 μL) of the resulting supernatant was centrifugally filtered through a Millipore 5-kDa cutoff filter (Merck Millipore) at 9100 × *g* and 4ºC for 120 min to remove proteins. The filtrate was evaporated, and the residue was resuspended in 50 μL of deionized water for mass spectrometric analysis. Metabolome analysis was performed through a facility service at Human Metabolome Technologies by capillary electrophoresis–TOF mass spectrometry for cation analysis and capillary electrophoresis–tandem mass spectrometry for anion analysis. Data are provided in Supplemental Tables 1 and 2. Hepatic citrate content was measured with 20 mg of liver tissue and a Citrate Assay Kit (Sigma-Aldrich, MAK057). In brief, the tissue was homogenized in Citrate Assay Buffer and the homogenate was centrifuged at 15,000 × *g* and 4ºC for 10 min to remove insoluble material. The resulting supernatant was depleted of protein with a Vivaspin 500 10,000 MWCO device (Sartorius, VS0101) and was then analyzed in triplicate, with the results expressed as nanomoles of citrate per gram of liver weight.

Measurement of hepatic glycogen, triglyceride, and cholesterol levels. Hepatic glycogen content was measured with 50 mg of liver tissue and a Glycogen Colorimetric Assay Kit (BioVision, K646-100). For determination of cholesterol and triglyceride levels, lipids were extracted from 40 to 50 mg of liver tissue with chloroform-methanol (2:1, vol/vol) according to the Folch method (9). A portion of the organic phase was dried, resuspended in 2-propanol, and analyzed for triglyceride or cholesterol with a Triglyceride E-test or Cholesterol E-test (Fujifilm Wako Pure Chemical), respectively. Samples were analyzed in triplicate and the results expressed as milligrams of lipid per gram of liver weight.

Determination of tissue glucose uptake, and liver PFK and glycogen phosphorylase activity. Mice that had been deprived of food for 16 h were injected intraperitoneally with a mixture of glucose and 2-DG at 0.5 and 1.5 g per kilogram of BW, respectively, dissolved in PBS. Liver and gastrocnemius muscle were excised rapidly at 30 min after 2-DG administration, and 2-deoxyglucose 6-phosphate levels in the tissue were determined with a

2-Deoxyglucose Uptake Measurement Kit (Cosmo Bio, MBR-PMG-K01). Hepatic PFK activity was measured with 40 mg of frozen liver tissue and a PFK Activity Colorimetric Assay Kit (BioVision, K776-100). Hepatic glycogen phosphorylase activity was measured with 50 mg of frozen liver tissue and a Glycogen Phosphorylase Assay Kit (Colorimetric) (Abcam, ab273271).

In vivo DNL assay. Mice were injected intraperitoneally with 35 μL per gram of BW of deuterated water (^2H_2O) (Sigma-Aldrich, 151882) or H_2O containing 0.9% NaCl and were maintained for 24 h with drinking water consisting of 8% enriched ^2H_2O or H_2O. They were deprived of food for the final 6 h of this labeling period and then killed by cervical dislocation for collection of plasma and the liver.

For ^2H enrichment analysis of liver palmitate, gas chromatography–mass spectrometry (GC-MS) analysis was performed as previously described (10), with minor modifications. Frozen liver tissue (25–50 mg) was pulverized with an automatic cryogenic pulverizer (Tokken), and Methylation Reagent A from a Fatty Acid Methylation Kit (Nacalai Tesque, 06482-04) and 1 μg of C23:0 fatty acid (Sigma-Aldrich, T6543) as an internal standard were directly added to the pulverized tissue. Total fatty acids (free and esterified) were methylated with the use of the Fatty Acid Methylation Kit, and the resulting fatty acid methyl esters (FAMEs) were purified with a Fatty Acid Methyl Ester Purification Kit (Nacalai Tesque, 06483-94). The FAME samples were evaporated, reconstituted in dichloromethane, and analyzed with a GCMS-QP2010 Ultra system (Shimadzu) equipped with a FAMEWAX fused silica capillary column (length of 30 m with an internal diameter of 0.25 mm and a film thickness of 0.25 μm) (Restek, 12497). Each sample (1 μL) was injected in splitless mode, with helium as the carrier gas at a flow rate of 45 cm/s (linear velocity). The injection port temperature was set at 250°C. The oven temperature was set to 40°C and maintained for 2 min before being increased to 140°, 200°, and 240°C at a rate of 20°, 11°, and 3°C/min, respectively, and it was then maintained at 240°C for 10 min. The interface and ion source temperatures were maintained at 250° and 200°C, respectively. The mass spectrometer was operated in electron impact ionization mode with an ionization energy of 70 eV. Selected ion monitoring was performed for specific ions. The ions monitored for methyl esterified palmitate with deuterium incorporation were those with a mass/charge (*m/z*) ratio of 270, 271, 272, 273, 274, 275, 276, 277, and 278. An *m/z* of 368 was used to monitor C23:0 fatty acid. Mass isotopomer (*m*) distribution was determined by weighted multiple linear regression analysis with the use of the *m/z* spectrum of methyl esterified palmitate from

liver samples of H_2O-loaded mice to remove anything other than ^2H from ^2H_2O that might add mass to palmitic acid (such as a contribution of the derivatizing agent or the natural abundance of ^{13}C, ^2H, and ^{18}O)(11). The calculated mass isotopomer distribution was expressed as molar fractions (m_0, m_1, m_2, etc., corresponding to the fractions of molecules containing 0, 1, 2, etc. ^2H substitutions, respectively). The relative amount of total tissue palmitate was determined by normalization with the AUC of C23:0 fatty acid.

The ^2H enrichment of water from plasma samples and standards was determined by deuterium acetone exchange (12). A 10-μL volume of sample or standard was mixed with 2 μL of 10 M NaOH and 5 μL of acetone in a GC vial and was incubated at room temperature for 4 h before analysis. Acetone was analyzed with the GCMS-QP2010 Ultra instrument equipped with a DB-17MS column (length of 15 m with an internal diameter of 0.25 mm and a film thickness of 0.15 μm) (Agilent, 122-4711). A portion (1 μL) of headspace acetone gas from the GC vial was injected in splitless mode, with helium as the carrier gas at a flow rate of 54.1 cm/s (linear velocity). The injection port temperature was set at 170°C. The oven temperature was set and held at 170°C for 2 min. The interface and ion source temperatures were maintained at 250° and 200°C, respectively. The mass spectrometer was operated in electron impact ionization mode with an ionization energy of 70 eV. Selected ion monitoring was adopted for specific ions. The ions monitored were those with an *m/z* of 58, 59, and 60. The AUC was used to calculate the ^2H enrichment of the samples by calibration with standards with a known ^2H mole fraction prepared by mixing H_2O and 99.9% ^2H_2O.

The fraction of newly synthesized fatty acids (FNS) was calculated as previously described (10). In brief, FNS is determined by the following equation: $FNS = ME/(N \times p)$, where ME is the average number of ^2H atoms incorporated per molecule [$ME = (m_1 \times 1) + (m_2 \times 2) + (m_3 \times 3) + \ldots$] and p is the ^2H enrichment of water calculated as above. N represents the number of exchangeable hydrogens and was calculated by the following equation: $m_2/m_1 = [(N - 1)/2] \times [p/(1 - p)]$. The relative amount of newly synthesized palmitate was determined by multiplying FNS by the relative amount of total tissue palmitate and then normalizing by tissue weight.

REFERENCES

1. Shibata H, Toyama K, Shioya H, Ito M, Hirota M, Hasegawa S, et al. Rapid colorectal adenoma formation initiated by conditional targeting of the Apc gene. *Science.* 1997;278(5335):120-3.

2. Holzenberger M, Lenzner C, Leneuve P, Zaoui R, Hamard G, Vaulont S, et al. Cre-mediated germline mosaicism: a method allowing rapid generation of several alleles of a target gene. *Nucleic Acids Res.* 2000;28(21):E92.

3. Yakar S, Liu JL, Stannard B, Butler A, Accili D, Sauer B, et al. Normal growth and development in the absence of hepatic insulin-like growth factor I. *Proc Natl Acad Sci U S A.* 1999;96(13):7324-9.

4. Balthasar N, Dalgaard LT, Lee CE, Yu J, Funahashi H, Williams T, et al. Divergence of melanocortin pathways in the control of food intake and energy expenditure. *Cell.* 2005;123(3):493-505.

5. Sakai M, Matsumoto M, Tujimura T, Yongheng C, Noguchi T, Inagaki K, et al. CITED2 links hormonal signaling to PGC-1alpha acetylation in the regulation of gluconeogenesis. *Nat Med.* 2012;18(4):612-7.

6. Matsumoto M, Ogawa W, Akimoto K, Inoue H, Miyake K, Furukawa K, et al. PKClambda in liver mediates insulin-induced SREBP-1c expression and determines both hepatic lipid content and overall insulin sensitivity. *J Clin Invest.* 2003;112(6):935-44.

7. Izumida Y, Yahagi N, Takeuchi Y, Nishi M, Shikama A, Takarada A, et al. Glycogen shortage during fasting triggers liver-brain-adipose neurocircuitry to facilitate fat utilization. *Nat Commun.* 2013;4:2316.

8. Sakai M, Tujimura-Hayakawa T, Yagi T, Yano H, Mitsushima M, Unoki-Kubota H, et al. The GCN5-CITED2-PKA signalling module controls hepatic glucose metabolism through a cAMP-induced substrate switch. *Nat Commun.* 2016;7:13147.

9. Folch J, Lees M, and Sloane Stanley GH. A simple method for the isolation and purification of total lipides from animal tissues. *J Biol Chem.* 1957;226(1):497-509.

10. Lee WN, Bassilian S, Ajie HO, Schoeller DA, Edmond J, Bergner EA, et al. In vivo measurement of fatty acids and cholesterol synthesis using D2O and mass isotopomer analysis. *Am J Physiol.* 1994;266(5 Pt 1):E699-708.

11. Lee WN, Byerley LO, Bergner EA, and Edmond J. Mass isotopomer analysis: theoretical and practical considerations. *Biol Mass Spectrom.* 1991;20(8):451-8.

12. Shah V, Herath K, Previs SF, Hubbard BK, and Roddy TP. Headspace analyses of acetone: a rapid method for measuring the 2H-labeling of water. *Anal Biochem.* 2010;404(2):235-7.

Unedited blot/gel images

Figure 1G

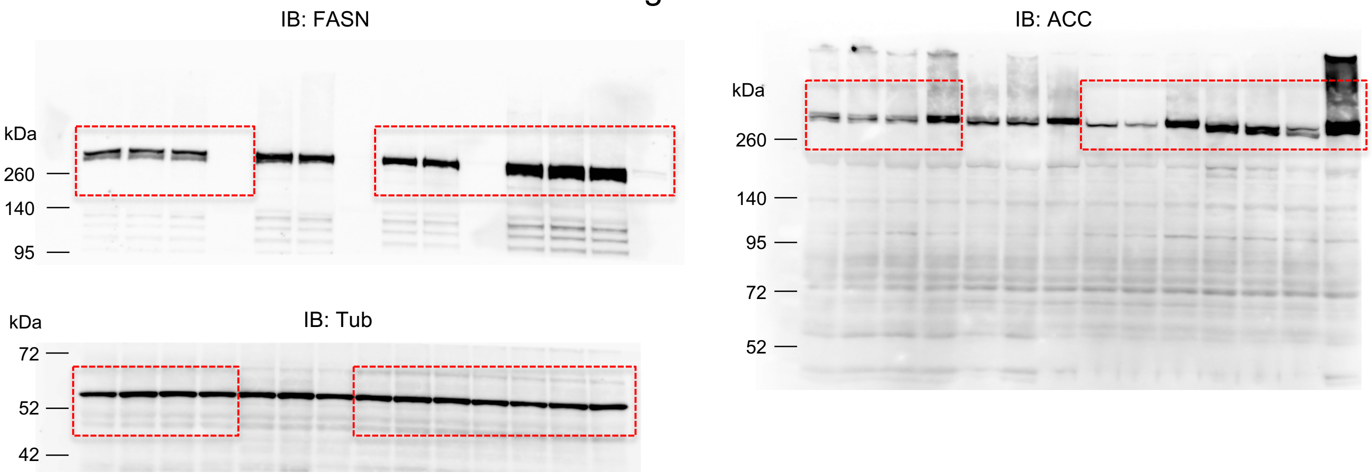

Figure 4G

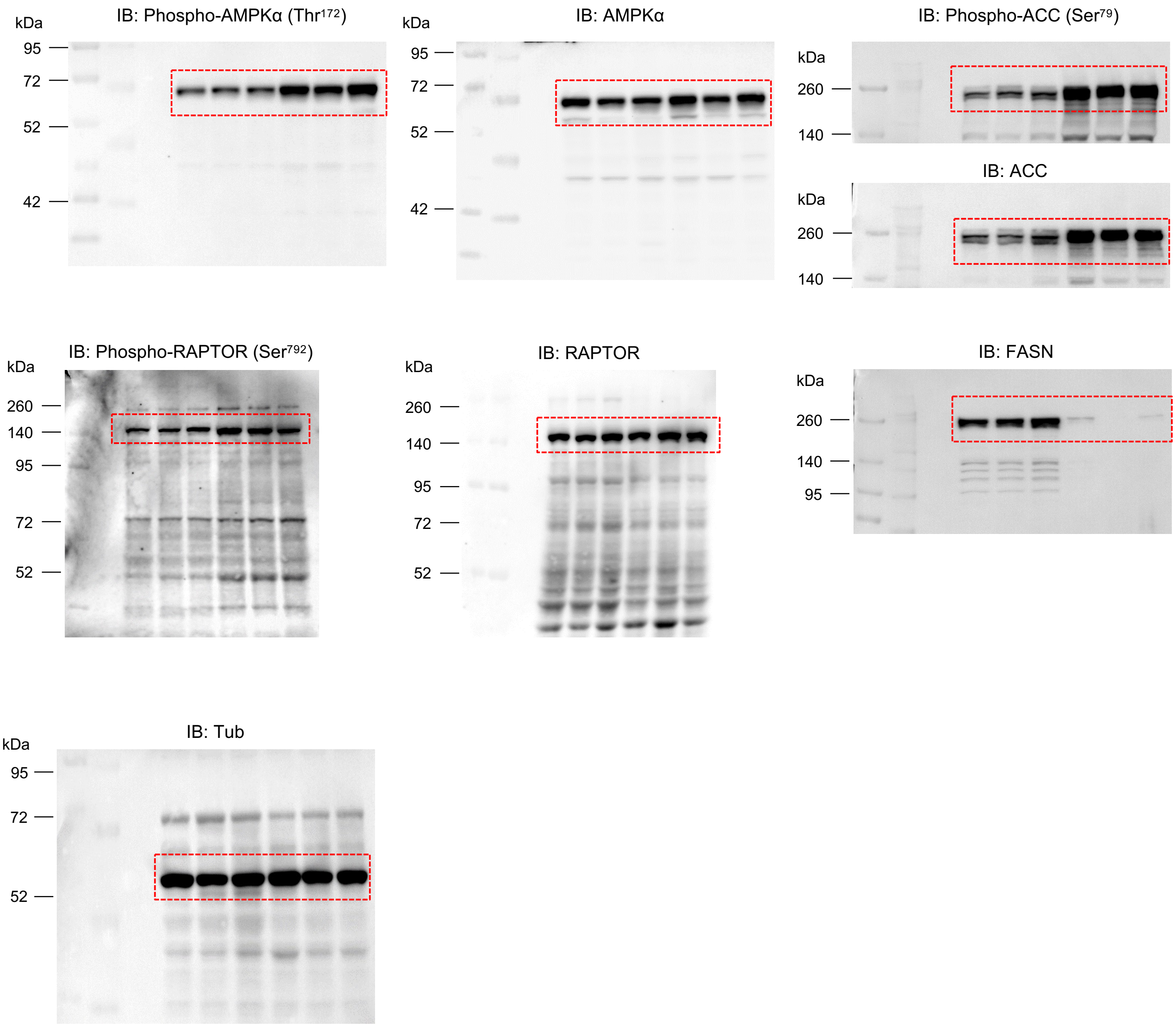

Figure 6L

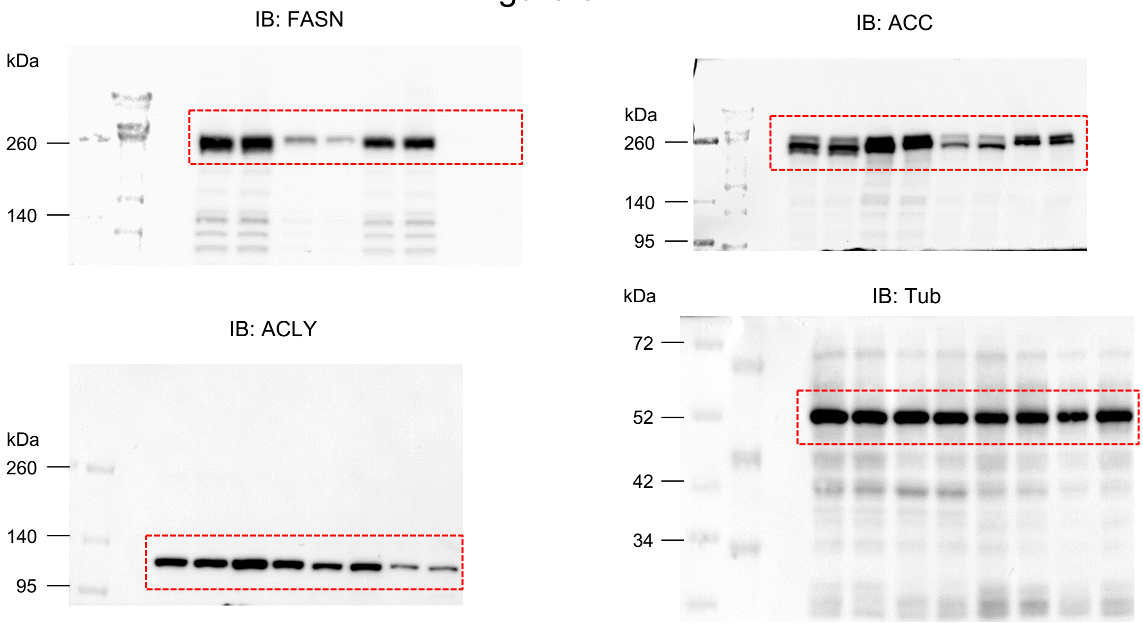

Figure 7E

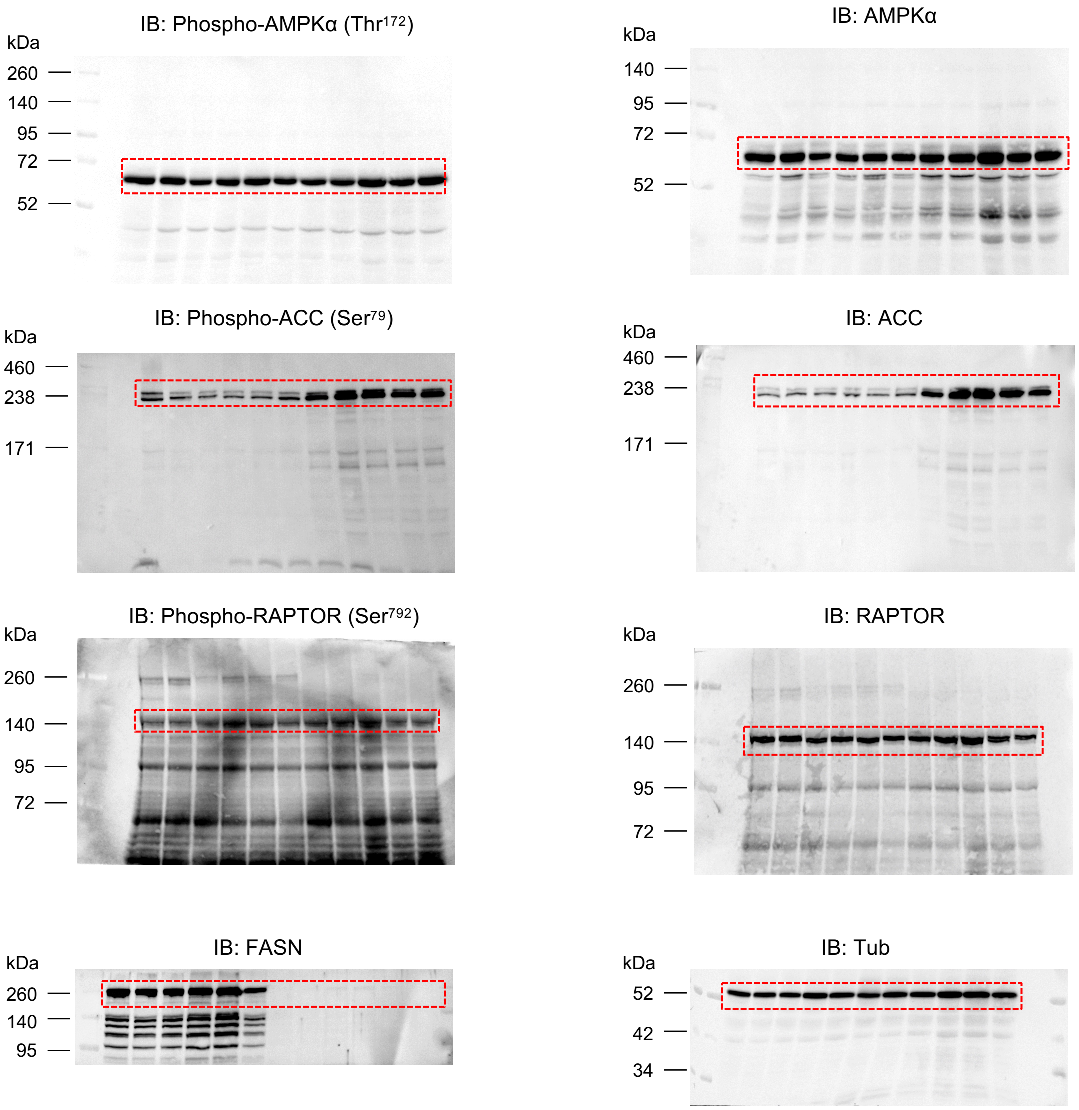

Figure 7F

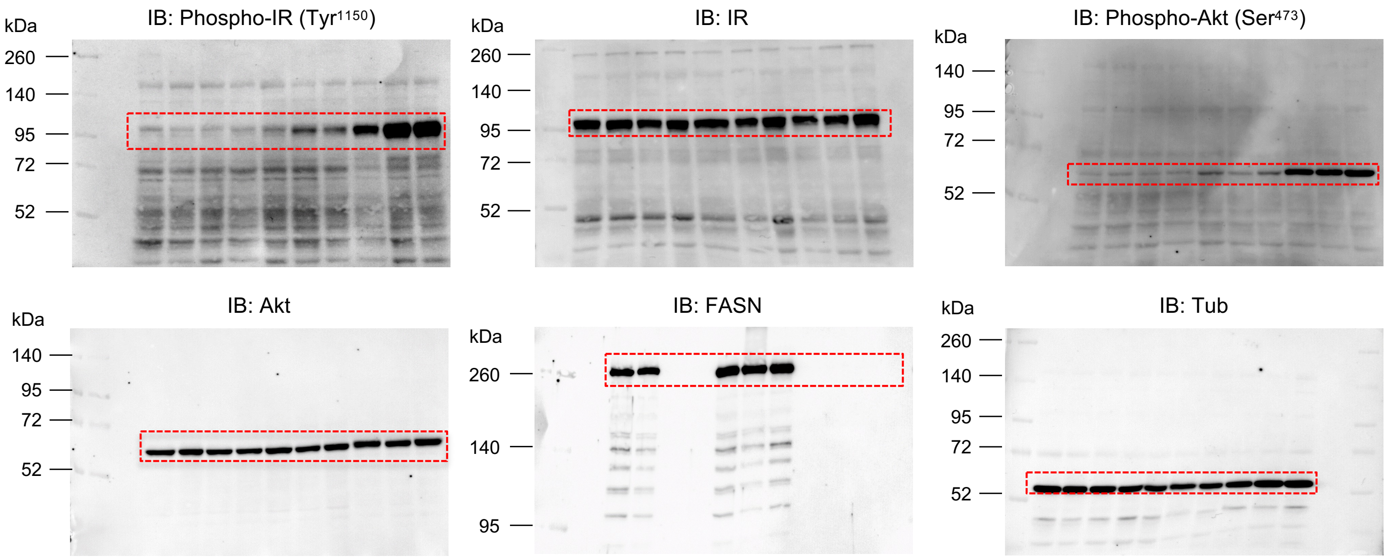

Supplemental Figure 1D

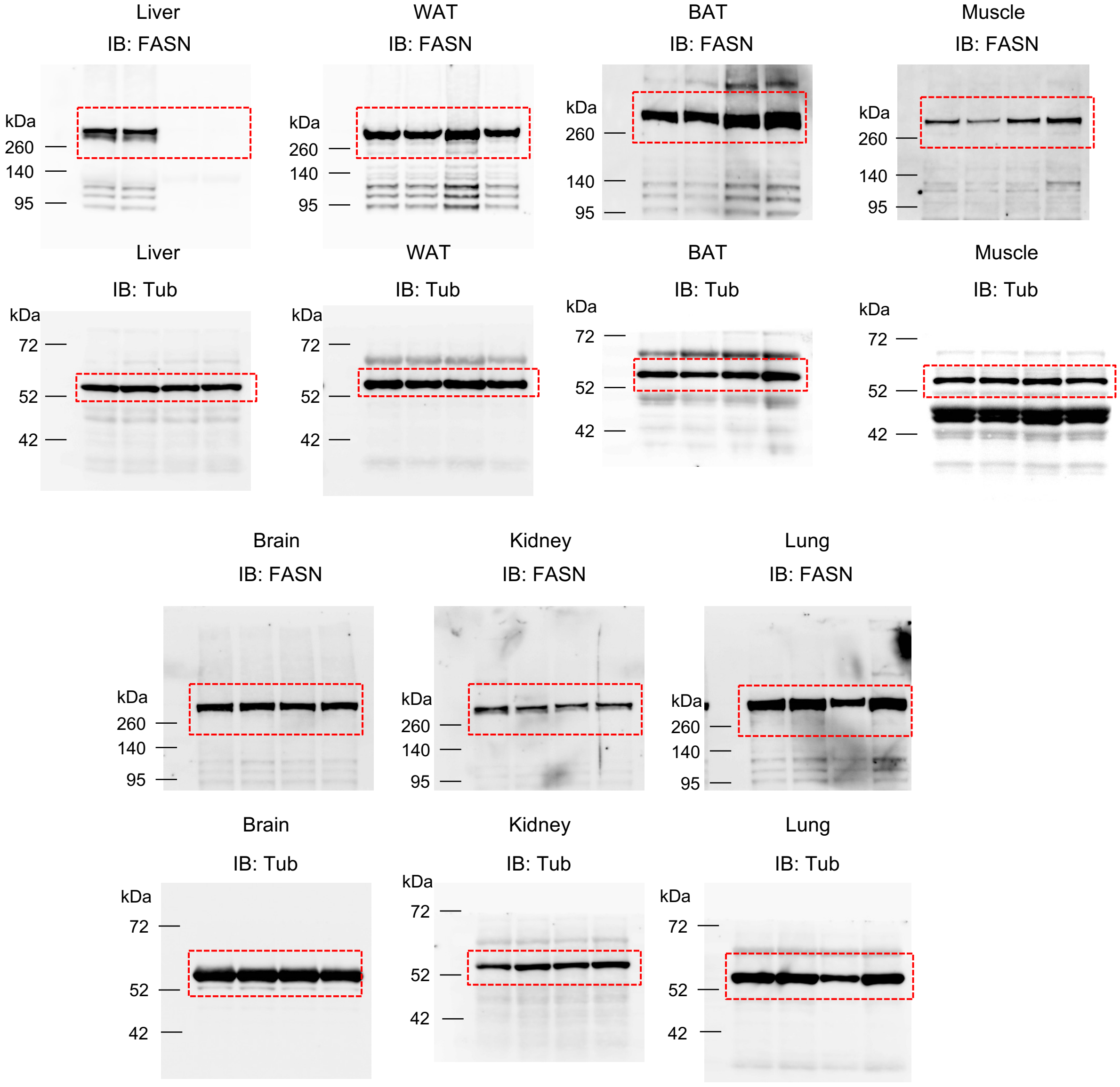

Supplemental Figure 4G

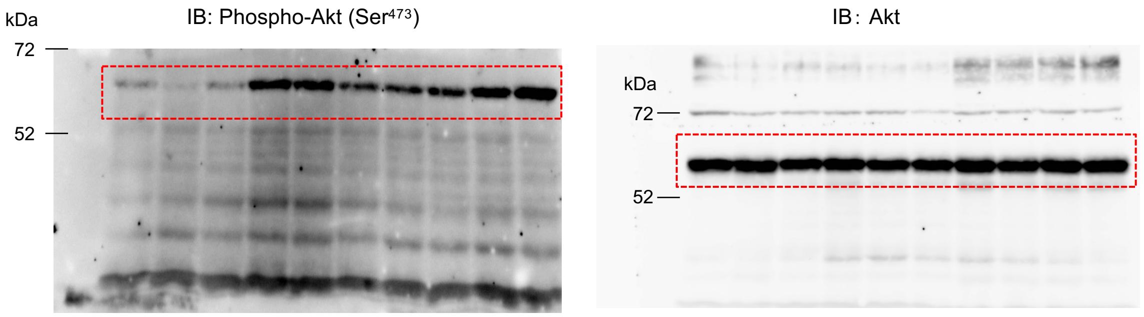

Supplemental Figure 4H

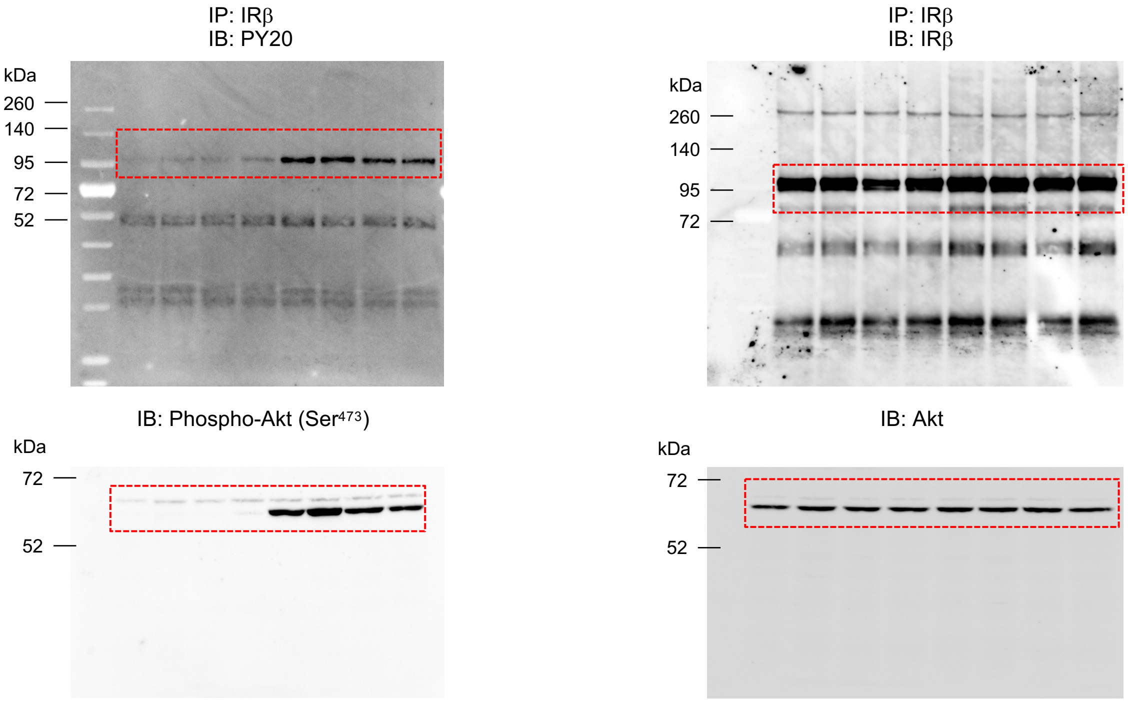

Supplemental Figure 5E

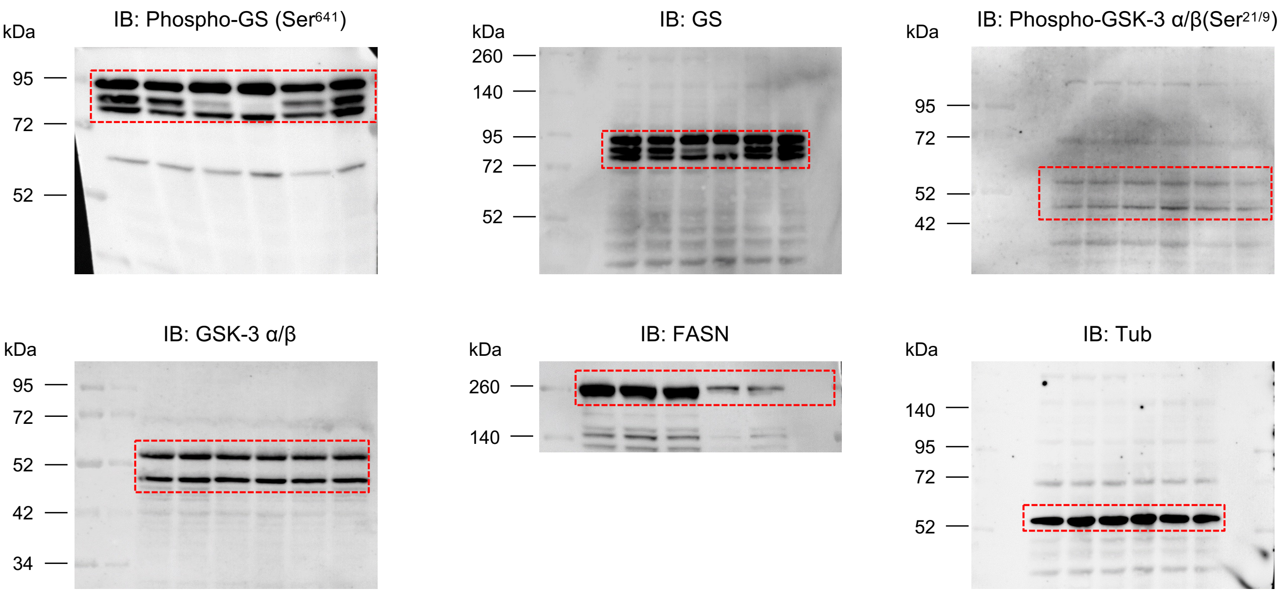

Supplemental Figure 6A

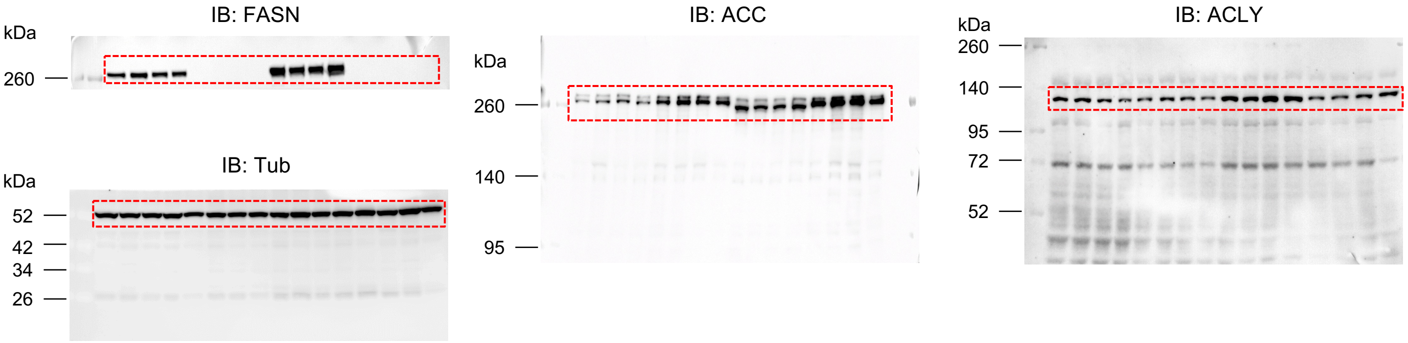

Supplement: Supplemental data [file jciinsight-8-161282-s106.pdf]
